# Sustainable Voltammetric Sensors Based on Rice-Husk-Derived Biosilica for Rapid, Low-Cost, and Selective Determination of Diuron in Water

**DOI:** 10.1021/acsomega.5c12781

**Published:** 2026-02-15

**Authors:** Roberta A. de Jesus, Gustavo V. de S. Santos, José A. do S. Costa, Katlin I. B. Eguiluz, Giancarlo R. Salazar-Banda, Zaine T. Camargo

**Affiliations:** † Department of Chemistry, Federal University of Sergipe, 49100-000 São Cristóvão, Sergipe, Brazil; ‡ Laboratory of Electrochemistry and Nanotechnology, Institute of Technology and Research (ITP), 49032-490 Aracaju, Sergipe, Brazil; § Process Engineering Graduate Program (PEP), 67896 Tiradentes University, 49032-490 Aracaju, Sergipe, Brazil; ∥ Study Group on Structured Nanomaterials, 245076 Federal University of Western Pará, 68040070 Santarém, Pará, Brazil

## Abstract

Diuron is a widely used herbicide that frequently contaminates soils and surface waters, posing risks to aquatic ecosystems and human health. Reliable and selective monitoring of diuron in environmental waters is therefore essential. In this work, we design carbon paste electrodes modified with rice-husk-derived biosilica and mesoporous MCM-41 as sustainable platforms for the voltammetric determination of diuron, and we benchmark their performance against commercial silica modifiers. Conventional carbon paste sensors (CPS) offer a versatile electroanalytical platform but often exhibit sluggish electron transfer and moderate sensitivity. The incorporation of biomass-derived silica promotes diuron preconcentration at the electrode–solution interface and markedly enhances the oxidation current. CPS containing 10% rice husk biosilica (rice husk_10_-CPS) and 10% rice-husk-derived MCM-41 (MCM-41_10_-CPS) exhibited the best performance, attributable to the increased electroactive surface area, mesopore-facilitated diffusion, and improved charge-transfer properties. Two linear dynamic ranges were obtained; the high-concentration range (0.08–2.0 mg L^–1^) was used to estimate limits of detection and quantification for rice husk_10_-CPS (LOD = 3.31, LOQ = 11.05, *R* ^2^ = 0.9925) and MCM-41_10_-CPS (LOD = 8.07, LOQ = 26.91, *R* ^2^ = 0.9979). The sensors retained their analytical response after 30 days of storage and remained reproducible for at least four reuse cycles. Interference tests in river water showed no measurable effect on the diuron signal from common pesticides and metal ions at 100-fold excess, and satisfactory recoveries (≈90–110%) were obtained by standard addition. These results demonstrate that rice-husk-derived silica is an effective, sustainable, and economical modifier for carbon paste electrodes, enabling robust voltammetric screening and determination of diuron in environmental water samples.

## Graphical abstract

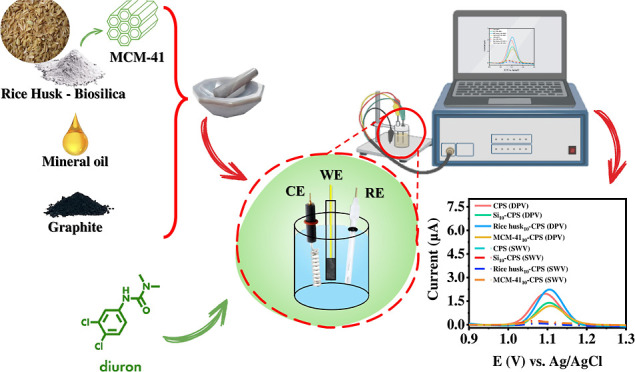

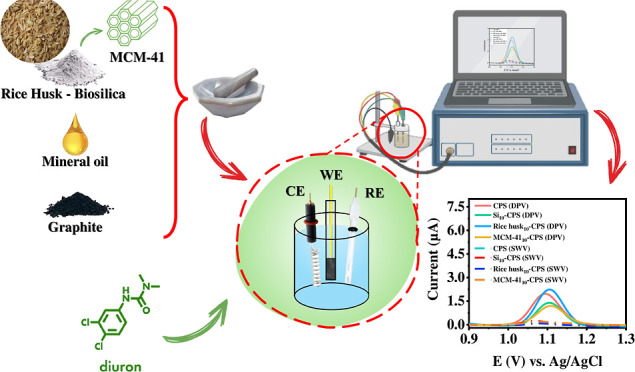

## 1 Introduction

Pesticide residues in foods and environmental matrices remain a global concern with documented ecotoxicological and human-health impacts, underscoring the need for analytical methods capable of their detection.^1,2^ Diuron (*N*′-(3,4-dichlorophenyl)-*N*,*N*-dimethylurea), a phenylurea herbicide introduced in the 1950s, persists in soils and waters and has been linked to adverse biological effects and potential human risks.^3^

Due to its environmental persistence and mobility, diuron is frequently found in rivers, often alongside other phenylureas such as isoproturon, exacerbated by diffuse runoff and insufficient wastewater treatment.^4^ Traditional methods for diuron determination, such as high-performance liquid chromatography,^5^ gas chromatography,^6^ and spectroscopic techniques,^7^ provide high sensitivity and accuracy. However, these methods require expensive instrumentation, laborious sample preparation, and specialized operators, which limits their use for routine or on-site measurements.^8^ Electroanalytical techniques, particularly differential pulse voltammetry using modified carbon paste electrodes, therefore provide a low-cost and sensitive approach with good selectivity and straightforward miniaturization potential for in situ monitoring of diuron.^9–11^

In electrochemical sensing, analyte concentration is quantified from changes in electrical response arising from interactions between the target species and the electrode interface, typically measured as current, potential, or impedance. In most configurations, the electrochemical cell comprises three electrodes: a working electrode, a reference electrode, and a counter (auxiliary) electrode. Common electroanalytical methods include potentiometry, cyclic voltammetry (CV), chronoamperometry, differential pulse voltammetry (DPV), square-wave voltammetry (SWV), electrochemical impedance spectroscopy (EIS), and linear sweep voltammetry.^12^

Electrochemical sensors for diuron employ diverse modifiers, including conjugated polymers, biorecognition elements, graphene derivatives, coordination compounds, and metal nanoparticles.^7–10^ Similarly, chemically modified carbon paste sensors (CPS) are particularly attractive owing to tunable composition, high surface area/porosity, and favorable charge transfer, but performance depends critically on the chosen modifier. Biomass-derived solids have emerged as sustainable CPS modifiers with synergistic physicochemical properties.^11–13^ A recurring limitation of conventional CPS is sluggish electron transfer; mesoporous silica can mitigate this by enlarging the electroactive area and favoring pore-assisted preconcentration.^14–18^

Agro-industrial residues are promising precursors for silica synthesis, aligning performance with environmental and economic goals. Among these residues, rice husk is particularly attractive because its ash is rich in amorphous silica (up to 99% by mass), enabling low-cost synthesis of nanostructured silica at subcrystallization temperatures (<700 °C). Compared with expensive molecular precursors such as tetraethyl orthosilicate, rice-husk-derived silica lowers materials cost and valorizes a waste stream, aligning analytical performance with environmental and economic goals.

Within the M41S family, MCM-41 combines high surface area, ordered hexagonal mesopores, and Lewis acidity that facilitate diffusion and interfacial mass transport, improving electrochemical responses.^18–20^ Its thermal/chemical robustness and surface renewability further support its use as a CPS modifier.^21^ Reported applications include enhanced electrocatalytic detection using MCM-41-based composites, with examples achieving low detection limits and wide dynamic ranges.^22–25^

Despite significant progress in the field, to date, no study has systematically compared rice-husk-derived silicas (biosilica and MCM-41) with commercial silica as CPS modifiers specifically for diuron determination. Cost-performance evaluations positioning waste-derived silicas as technically competitive options are likewise limited. We therefore hypothesize that incorporating rice-husk-derived mesoporous silicas into CPS increases peak currents and decreases the limits of detection and quantification for diuron relative to commercial silica, owing to the enlarged electroactive area and pore-mediated preconcentration. Such improvements are particularly relevant for environmental monitoring programs, where sensitive, low-cost sensors are needed to track diuron contamination in surface and drinking-water sources.^26–29^

Here, we develop CPS modified with silica additives prepared from rice husk (biosilica and MCM-41) and, for comparison, commercial silica gel, and evaluate their performance for diuron detection by DPV. We optimize electrode composition and operating parameters (modifier loading and structure, scan settings, detection mode, and supporting-electrolyte pH and identity) and assess sensitivity, selectivity, stability, reusability, cost per analysis, and performance in river water samples. We aim to couple the sustainability benefits of biomass-derived silica with the analytical advantages of ordered mesoporosity for sensitive, selective, and cost-effective diuron monitoring.

## 2 Experimental Section

### 2.1 Materials

Rice husk was supplied by the Brazilian Agricultural Research Corporation (São Paulo, Brazil). Commercial silica gel was purchased from Merck. Diuron, graphite powder, potassium chloride (KCl), potassium ferricyanide, potassium ferrocyanide, cetyltrimethylammonium (CTAB), sodium hydroxide (NaOH), hydrochloric acid (HCl), phosphoric acid, acetic acid, boric acid, dibasic sodium phosphate, monobasic sodium phosphate, sodium chloride (NaCl), aqueous ammonia (25 wt% NH_3_), 4-nitrophenol, lead­(II) chloride (PbCl_2_), iron­(II) sulfate heptahydrate (FeSO_4_·7H_2_O), mercury­(II) sulfate (HgSO_4_), copper­(II) sulfate pentahydrate (CuSO_4_·5H_2_O), acetaminophen, triclosan, ametryn, carbaryl, and triflularin were obtained from Sigma-Aldrich (≥99% purity). All reagents were of analytical grade. Solutions were prepared using ultrapure water.

Britton–Robinson (BR) buffer was prepared by mixing phosphoric, acetic, and boric acids, each at 0.1 mol L^–1^, and adjusting the pH with NaOH or HCl as specified. Phosphate buffer PBS-1 (0.2 mol L^–1^, pH 8) was prepared by dissolving 0.08 g of Na_2_HPO_4_, 0.04 g of NaH_2_PO_4_, and 1.05 g of KCl in 100 mL of ultrapure water. Phosphate buffer PBS-2 (0.2 mol L^–1^, pH 4) was prepared by dissolving 1.42 g of Na_2_HPO_4_ and 2.4 g of NaH_2_PO_4_ in 100 mL of ultrapure water. The pH of all solutions was adjusted to the desired value using 3 mol L^–1^ NaOH and 3 mol L^–1^ HCl solutions.

### 2.2 Rice Husk Ash Preparation and MCM-41 Synthesis

Silica was extracted from rice husk following Costa and Paranhos.^30^ Briefly, husk from the Agulhinha variety was calcined at 700 °C for 2 h to obtain rice husk ash (RHA). Mesoporous MCM-41 was synthesized hydrothermally according to Costa et al.^31^ 3 g of CTAB was dissolved in 24 mL of concentrated aqueous ammonia solution, followed by the addition of 54 mL of deionized water and 50 mL of sodium silicate solution obtained from rice husk ash as a silica source. Afterward, the mixture was stirred at room temperature for 24 h (aging), transferred to a Teflon-lined stainless-steel autoclave, and heated at 100 °C for 48 h. The solid was recovered by filtration, washed thoroughly with deionized water to remove residual surfactant and salts, and air-dried at room temperature for 12 h. Surfactant removal was then performed following Costa et al.^32^ to obtain template-free MCM-41.

### 2.3 Sensor Preparation

Unmodified CPS were prepared by hand-mixing graphite powder (0.14 g) with mineral oil (0.06 g) in an agate mortar for approximately 10 min until a homogeneous paste was obtained. The paste was packed into a polypropylene tube (exposed geometric area 0.18 cm^2^), and the surface was smoothed on cellulose filter paper to yield the unmodified CPS.

Modified sensors were prepared by keeping the binder content constant and partially replacing graphite with a silica-based modifier at 10 or 20 wt % relative to the total paste mass (0.20 g). For the 10 wt % formulation, graphite (0.12 g), mineral oil (0.06 g), and modifier (0.02 g) were mixed. For the 20 wt % formulation, graphite (0.10 g), mineral oil (0.06 g), and modifier (0.04 g) were used. The modifiers were commercial silica gel, rice-husk-derived biosilica, and rice-husk-derived MCM-41. Mixing, packing, and surface finishing followed the procedure for the unmodified CPS. The electrodes were designated by modifier and loading: Si_10_-CPS, Rice Husk_10_-CPS, and MCM-41_10_-CPS (10 wt %); Si_20_-CPS, Rice Husk_20_-CPS, and MCM-41_20_-CPS (20 wt %).

### 2.4 Instrumentation and Electrochemical Characterization

Electrochemical measurements were carried out in a 25 mL three-electrode cell using the CPS or its modified variants (CPS, Si_10_-CPS, rice Husk_10_-CPS, MCM-41_10_-CPS, Si_20_-CPS, rice Husk_20_-CPS, or MCM-41_20_-CPS) as the working electrode, a platinum wire as the counter electrode, and an Ag/AgCl (3.0 mol L^–1^ KCl) reference electrode. Supporting electrolytes included 1.0 mmol L^–1^ ferri/ferrocyanide ([Fe­(CN)_6_]^3–/4–^) in 1 mol L^–1^ KCl, BR buffer, and two phosphate-buffered saline solutions (PBS1 and PBS2). Electrochemical experiments were performed using a Metrohm Autolab potentiostat/galvanostat (model AUT85367) controlled by NOVA 2.1.8 software.

The CV in [Fe­(CN)_6_]^3–/4–^ was recorded from −0.5 to +1.0 V, with scan rates from 25 mV s^–1^ to 200 mV s^–1^, to estimate the effective electroactive surface area of each electrode. Electrochemical impedance spectroscopy (EIS) was carried out in the same redox probe, over 0.1 to 1 × 10^5^ Hz with a small-signal AC amplitude (typically 5–10 mV rms), to characterize charge-transfer and mass-transport processes. The DPV for the [Fe­(CN)_6_]^3–/4–^ redox couple was acquired at 20 mV s^–1^ from 0.0 to +0.6 V.

The intrinsic electrochemical behavior of the sensors was further examined by CV in BR buffer (0.1 mol L^–1^, pH 3.0) from +0.7 to +1.2 V at scan rates of 25–300 mV s^–1^. Diuron (2 mg L^–1^) was quantified by DPV using a pulse time of 10 ms, pulse amplitude of 100 mV, and scan speed of 20 mV s^–1^ over +0.9 V to +1.3 V. Unless otherwise stated, all potentials are reported versus Ag/AgCl (3.0 mol L^–1^ KCl) at ambient laboratory temperature.

### 2.5 Physicochemical Characterization

Thermogravimetric analysis (TGA) was carried out using a Shimadzu DTG-60H analyzer (Kyoto, Japan) from 30 to 900 °C under flowing nitrogen (20 mL min^–1^) at a heating rate of 10 °C min^–1^. X-ray diffraction (XRD) patterns were recorded on a Shimadzu XRD-7000 diffractometer employing Co Kα radiation (λ = 1.789 Å) at 40 kV and 20 mA over 2θ = 5–80° with a scan rate of 0.5° min^–1^. Fourier transform infrared (FTIR) spectra were acquired on a PerkinElmer Spectrum Two spectrometer in the range 4000–400 cm^–1^ (resolution of 4 cm^–1^, 20 scans) using KBr pellets. Prior to analysis, rice husk-derived samples and MCM-41 were degassed at 140 °C for 4 h.

Textural properties (specific surface area and pore size distribution) were obtained by N_2_ physisorption. Specific surface area was calculated by the Brunauer–Emmett–Teller (BET) method, and mesopore size distributions were derived by the Barrett–Joyner–Halenda (BJH) method from the desorption branch. For transmission electron microscopy (TEM), powders were dispersed in isopropanol, sonicated for 15 min, and a drop of the suspension was deposited on a carbon-coated copper grid. After drying at room temperature, images were recorded on a JEOL JEM-1400 Plus operated at 120 kV.

## 3 Results and Discussion

### 3.1 Characterization of Rice-Husk-Derived Silica and MCM-41

Figure 1a shows the FTIR spectra of rice-husk-derived and MCM-41. The broad absorption band near 3470 cm^–1^ for MCM-41 and 3316 cm^–1^ for rice-husk silica arises from O–H stretching vibration of surface silanols (Si–OH) and physisorbed water molecules from moisture in the samples. The band near 1635 cm^–1^ for MCM-41 and 1644 cm^–1^ for rice-husk silica belongs to the H–O–H bending mode of adsorbed water, consistent with moisture retained on the silica surface.^33,34^ Weak features at around 2994 and 2886 cm^–1^ in the rice-husk silica are attributed to C–H stretching from trace saturated aliphatic compounds remaining after heat treatment (e.g., cellulose/hemicellulose residues).^35^ The small band at 968 cm^–1^ for MCM-41 is related to the stretching of the tetrahedrally coordinated Si–OH groups. The strong bands around ≈1100, ≈800, and ≈462 cm^–1^ in both materials are ascribed, respectively, to asymmetric and symmetric Si–O–Si stretching and to Si–O–Si bending modes of the siloxane network.^33,34^

**Figure 1:**
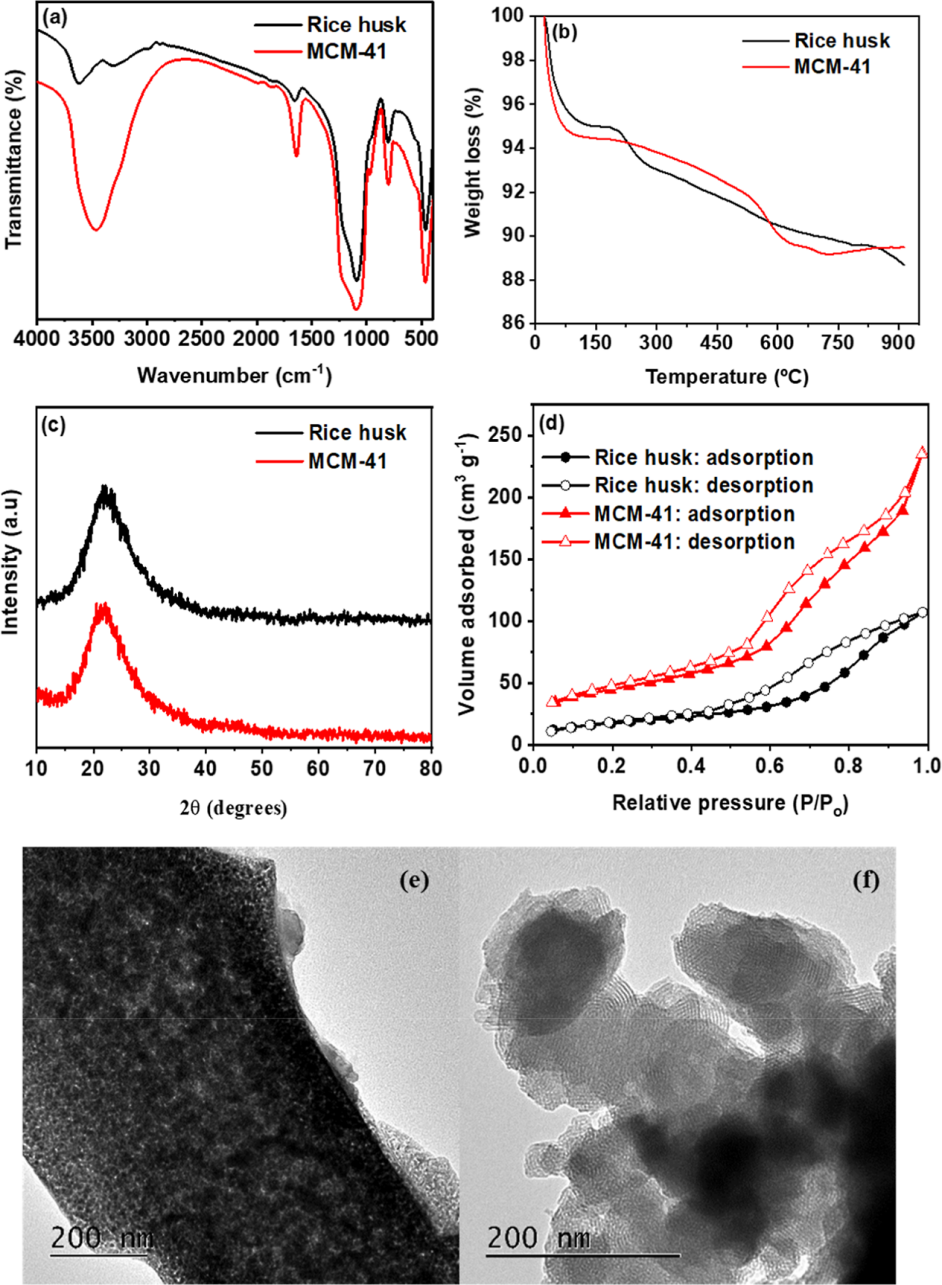
(a) FTIR spectra; (b) thermogravimetric (TG) profiles; (c) X-ray diffraction (XRD) patterns; (d) N_2_ adsorption–desorption isotherms of rice husk and MCM-41; (e) TEM micrograph of rice husk; and (f) TEM micrograph of MCM-41.

Thermogravimetric profiles are displayed in Figure 1b. The rice-husk silica exhibits an initial mass loss of ≈6% between ≈32 and 200 °C, attributed to desorption of physisorbed water, consistent with its hydrophilic surface. A subsequent ≈3% loss from ≈200 to 550 °C likely reflects the removal of residual organics originating from lignocellulosic precursors (hemicellulose, cellulose, and lignin undergo thermal decomposition over ≈200–360 °C).^30^ At temperatures above 550 °C under nitrogen, gradual mass changes are consistent with further removal/condensation of surface species (e.g., dehydroxylation) rather than oxidative processes. Above 700 °C, a decarbonization process occurs, associated with the elimination of unburned carbon residues and the thermal transformation of the remaining mineral constituents.^36^ For MCM-41, a ≈6% loss between ≈30 and 125 °C corresponds to physically adsorbed water on its surface or in its pores. At 230 and 650 °C, there is a slight mass loss that may be associated with the progressive dehydroxylation of silanols present in the silica framework.^37,38^

The XRD patterns for rice-husk–derived silica and MCM-41 (Figure 1c) exhibit a broad halo between 10° and 40°, centered at 2θ ≈ 22°, characteristic of amorphous silica (JCPDS 47-0715). The absence of sharp diffraction peaks confirms the amorphous nature of the materials. This profile matches the pattern of commercial silica gel, which also has an amorphous structure,^19^ as well as reports for biomass-derived amorphous silicas.^17^ Furthermore, the broad diffraction peak width suggests the presence of nanoscale pores, a feature also observed in studies by Hoang and collaborators for nanosilica.^39^ The profile for MCM-41 corroborates that the channel walls of the mesoporous structure are amorphous in nature, as already reported in the literature.^40–42^

Nitrogen physisorption isotherms (Figure 1d) for both materials are consistent with mesoporosity and exhibit type IV behavior with a narrow hysteresis loop, in line with IUPAC descriptions for cylindrical mesopores.^41^ The hysteresis loop is associated with capillary condensation, indicative of regular mesopores, as reported in the literature for the ordered hexagonal channels of MCM-41.^43,44^ Textural parameters (BET surface area, total pore volume, and BJH pore diameter) for rice-husk silica, MCM-41, and commercial silica gel are summarized in Table 1. TEM micrographs (Figure 1e,f) reveal porous morphologies for both samples, in agreement with the N_2_ physisorption data. However, MCM-41 shows domains of interconnected particles with evidence of local ordering, corroborating an ordered mesoporous architecture and supporting the adsorption–desorption analysis.

**Table 1:** Textural and Structural Properties of Rice Husk, MCM-41, and Commercial Silica Gel

sample	*S*_BET_ ^a^ (m^2^ g^–1^)	*V*_BJH_ ^b^ (cm^3^ g^–1^)	*D*_BJH_ ^c^ (nm)	ref
rice husk	158.95	0.35	3.17	this work
MCM-41	287.40	0.82	4.51	this work
commercial silica gel	370.80	0.87	3.98	^19^

a *S*_BET_, specific surface area determined by the BET method.

b V_BJH_, pore volume from BJH analysis.

c D_BJH_, pore diameter from BJH analysis.

The physicochemical properties of the commercial silica gel (99.8% purity) were previously reported by de Jesus et al.^19^ The FTIR spectra display bands at the expected wavenumbers across the analyzed range, consistent with siloxane (Si–O–Si) and silanol (Si–OH) vibrations, thereby corroborating the silica signatures in rice husk and MCM-41. Likewise, the XRD patterns show the broad amorphous halo characteristic of silica; the commercial silica and the rice-husk-derived sample exhibit comparable features, further supporting the presence of amorphous silica in rice husk.

Therefore, this study aimed to develop a low-cost carbon-paste electrode for diuron detection by incorporating silica derived from agro-industrial rice-husk waste as a modifying phase. Notably, rice husk offers a pronounced economic advantage: its cost (5.61 USD kg^–1^) is far lower than that of commercial silica gel (115 USD kg^–1^) and also below the cost of producing MCM-41 by conventional routes.

Although silica is electrically insulating, it can improve the electrochemical performance of composite electrodes when used as an additive by providing structural and interfacial benefits rather than direct electronic conduction. In particular, silica can increase the accessible surface area and tune the pore architecture, which may expose more electroactive sites and improve electrolyte wettability and ion transport. In addition, silica can promote a more uniform dispersion of the conductive phase and active components within the electrode matrix, which can improve percolation of the conductive network and yield more reproducible charge-transfer behavior across the composite.^45–47^

### 3.2 Electrochemical Characterization of CPS, Si_10_-CPS, Si_20_-CPS, Rice Husk_10_-CPS, Rice Husk_20_-CPS, MCM-41_10_-CPS, and MCM-41_20_-CPS

Figure 2 compiles cyclic voltammograms for CPS, Si_20_-CPS, rice husk_20_-CPS, and MCM-41_20_-CPS, and Figure S1 for Si_10_-CPS, rice husk_10_-CPS, and MCM-41_10_-CPS, all recorded at the scan rates of 25, 50, 75, 100, 125, 150, 175, and 200 mV s^–1^. Figures 2a–d and S1 display the expected reversible ferri/ferrocyanide couple, with the anodic peak near +0.29 to +0.35 V and the cathodic counterpart at +0.19 to +0.22 V.^48,49^ The modified electrodes, particularly Si_20_-CPs and rice husk_20_-CPS, exhibit higher peak currents for the [Fe­(CN)_6_]^3–/4–^ redox couple than the unmodified CPS. This enhancement is consistent with a larger electroactive surface area imparted by the modifiers, which facilitates charge transfer and improves sensor sensitivity.^50^ As the scan rate increases, peak currents rise with the anticipated υ^1/2^ dependence, accompanied by moderate peak broadening and a slight increase in peak separation (Δ*E*_p_), reflecting uncompensated resistance and finite charge-transfer kinetics at higher sweep speeds.

**Figure 2:**
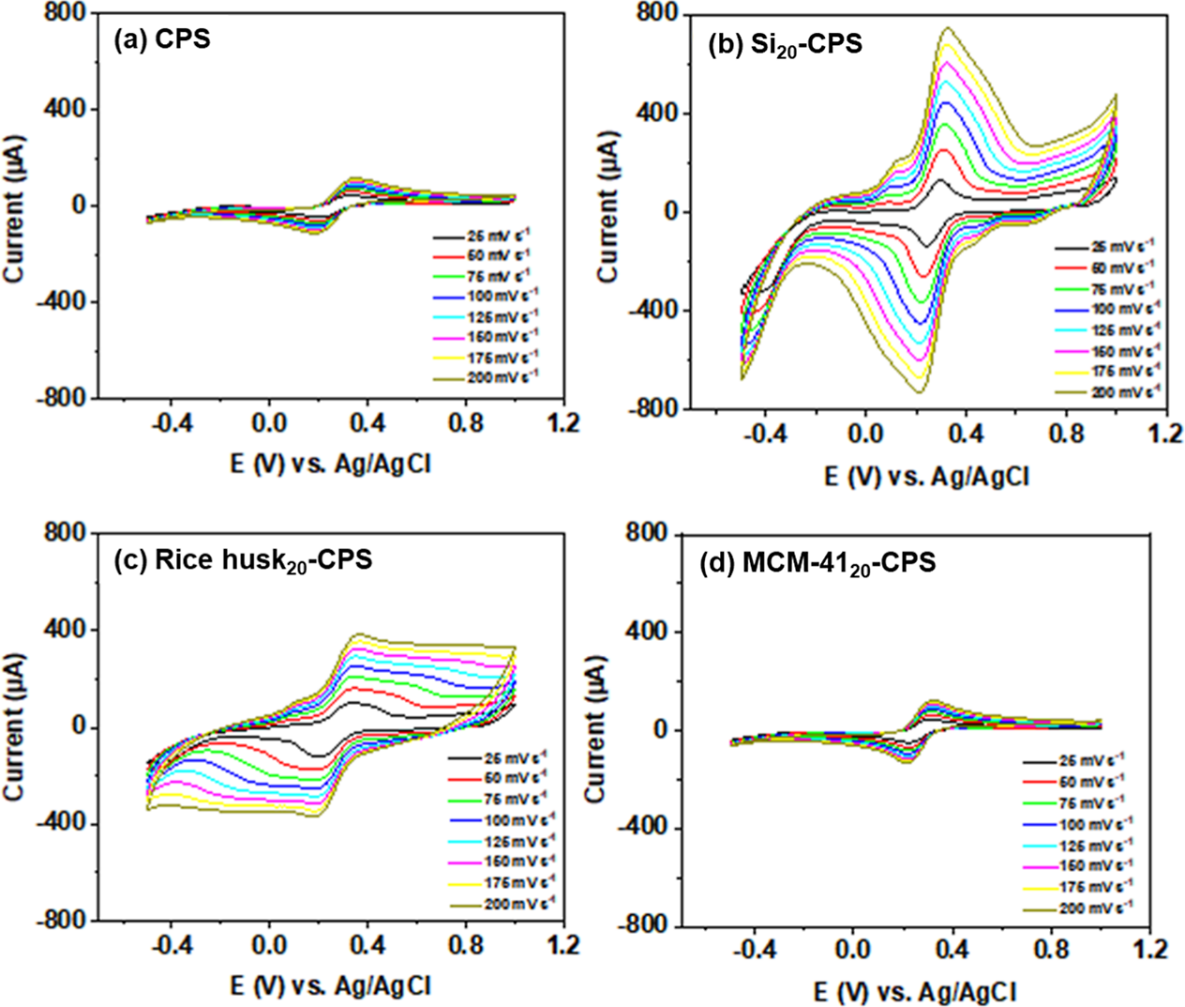
Cyclic voltammograms recorded at different scan rates (25–200 mV s^–1^) for (a) CPS, (b) Si_20_-CPS, (c) rice husk_20_-CPS, and (d) MCM-41_20_-CPS electrodes in 1.0 mmol L^–1^ [Fe­(CN)_6_]^3–^/[Fe­(CN)_6_]^4–^solution.

The [Fe­(CN)_6_]^3–^/[Fe­(CN)_6_]^4–^ couple was used as a standard probe to estimate the electroactive surface area (ECSA). CVs were acquired at varying scan rates, and plots of peak current (*I*_p_) versus (υ^1/2^) were constructed. The slopes were fitted to the Randles–Sevcik equation for a reversible diffusion-controlled process to calculate the effective area of each electrode.^51^

All electrodes exhibited electroactive areas larger than their geometric area (0.18 cm^2^). Specifically, the electroactive areas determined were: CPS (0.36 cm^2^), Si_10_-CPS (0.50 cm^2^), Si_20_-CPS (1.86 cm^2^), rice husk_10_-CPS (0.36 cm^2^), rice husk_20_-CPS (1.04 cm^2^), MCM-41_10_-CPS (0.49 cm^2^), and MCM-41_20_-CPS (0.38 cm^2^). This increase is due to the increased surface roughness and porosity of these materials, which contribute to their improved electrochemical performance discussed below.

Plots of peak current versus ν^1/2^ in Figure S2a–g were linear for all electrodes, indicating diffusion-controlled charge transfer for the [Fe­(CN)_6_]^3–/4–^ redox couple.^52,53^ Relative to CPS, the modified electrodes showed larger slopes in the *I*_p_–ν^1/2^ regressions (except for rice husk_10_-CPS), which is consistent with higher effective area and/or facilitated mass transport at the electrode–solution interface in the presence of the modifiers.

The anodic-to-cathodic peak current ratios (*I*_pa_/*I*_pc_) were close to unity for all electrodes (Table S1), indicating near-reversible behavior under the conditions used.^54,55^ All sensors exhibited a Δ*E*_p_ greater than 59 mV n^–1^. However, Δ*E*_p_ decreased from CPS to most modified electrodes, especially for Si_10_-CPS, Si_20_-CPS, and MCM-41_20_-CPS, implying faster apparent electron-transfer kinetics within a quasi-reversible regime. These observations suggest that silica-based modifiers enhance the interfacial process, likely through a combination of higher electroactive area and modified interfacial microenvironment. These outcomes agree with prior reports in which CPS modified with commercial silica gel or biosilica (e.g., from sugar cane ash) improved ferri/ferrocyanide responses.^19^ The literature has attributed such behavior in nanostructured silicas to electron transfer assisted by increased roughness, mesoporosity, and possible hopping-type pathways across silica–carbon interfaces.^18,19,56,57^ In this context, to further probe interfacial kinetics, we conducted electrochemical impedance spectroscopy (EIS), which provides complementary information on charge-transfer resistance and mass-transport characteristics.

The DPV measurements in [Fe­(CN)_6_]^3–/4–^ solution were performed to analyze the electrochemical activity of the electrodes (Figure S3). All modified electrodes showed higher anodic peak current intensities than the unmodified CPS during [Fe­(CN)_6_]^3–/4–^ oxidation, suggesting more efficient electron-transfer reactions at the composite interfaces. The superior performance of the Si_20_-CPS, rice husk_20_-CPS, and MCM-41_20_-CPS electrodes can be attributed to the incorporation of the silica-based modifiers into the CPS matrix, which alters the graphite content and improves the interfacial properties of the composite. The modest positive shifts in peak potential observed for these modified electrodes relative to CPS (CPS = +0.24 V, Si_20_-CPS = +0.25 V, rice husk_20_-CPS = +0.27 V, and MCM-41_20_-CPS = +0.25 V) are consistent with changes in composite composition and microstructure upon addition of the silica modifiers. In contrast, Si_10_-CPS, rice husk_10_-CPS, and MCM-41_10_-CPS showed peak potential similar to CPS (+0.24 V). The peak-current enhancements observed for all modified electrodes reinforce the beneficial role of the silica phases in optimizing the electrochemical response of carbon-paste electrodes toward this outer-sphere redox couple.^52,53^

The comparative DPV responses were consistent with electroactive surface-area estimates obtained by cyclic voltammetry: Si_20_-CPS and rice husk_20_-CPS presented larger surface areas than Si_10_-CPS and rice husk_10_-CPS, whereas MCM-41_20_-CPS exhibited a smaller surface area than MCM-41_10_-CPS under the conditions tested (Figures 2, S2 and S3). For subsequent studies, we selected the 10%-modifier electrodes because they maintained high analytical signals while reducing background currents (Figure S3), thereby providing a practical balance between sensitivity and operational robustness.

EIS was used to study the interface properties of different electrodes during the modification process. Nyquist plots in Figure 3 revealed a large semicircle for CPS, yielding a charge-transfer resistance *R*_ct_ of 1560 Ω, followed at lower frequencies by a linear tail associated with semi-infinite diffusion (Warburg behavior). This response indicates sluggish electron-transfer kinetics for [Fe­(CN)_6_]^3–/4–^ at the unmodified paste. In contrast, CPS, Si_10_-CPS, rice husk_10_-CPS, and MCM-41_10_-CPS have markedly smaller high-frequency semicircles, with Rct values of 130.4, 131.2, and 240 Ω, respectively, consistent with accelerated interfacial charge transfer relative to CPS.^53^ The data were fitted using a Randles-type equivalent circuit (inset in Figure 3), where *R*_s_ represents the solution resistance, *R*_ct_ the charge-transfer resistance, *C*_dl_ the double-layer capacitance, and *W* the Warburg element, confirming that semi-infinite diffusion dominates at low frequencies.^58^ These EIS results corroborate the CV/DPV data (Figure 2), underscoring the functional role of silica-based modifiers derived from agro-industrial waste in enhancing the electrode response of CPS electrodes.

**Figure 3:**
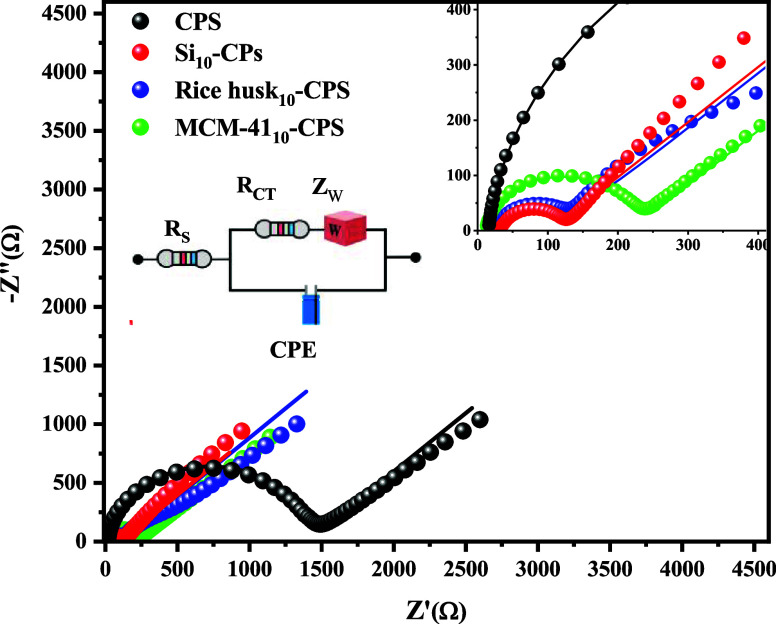
Nyquist diagrams obtained by EIS for CPS, Si_10_-CPS, rice husk_10_-CPS, and MCM-41_10_-CPS electrodes recorded in a 1 mmol L^–1^ [Fe­(CN)_6_]^3–/4–^ electrolyte solution.

We attribute the improvements not to intrinsic conductivity of silica, but to composite-level effects such as (i) formation of a porous network that increases electroactive area and facilitates electrolyte access; (ii) improved exposure and percolation of graphite particles within the paste; and (iii) a modified microenvironment at the silica–carbon–electrolyte interface that lowers the effective barrier for heterogeneous electron transfer. Similar enhancements have been reported for CPS modified with commercial silica gel and biosilica from sugar cane ash.^19,56,59,60^

### 3.3 Electrochemical Behavior of CPS, Si_10_-CPS, Rice Husk_10_-CPS, and MCM-41_10_-CPS toward Diuron

#### 3.3.1 Effect of Electrolyte Solution, pH, and Sweep Rate

We investigated the electrochemical response of diuron by DPV in BR buffer (Figure S4) and phosphate buffer (Figure S5) at different pH values. For the BR buffer, we examined pH 2–6; for PBS, we tested pH 6 and 7 in one formulation and pH 2–4 in another. The voltammograms in Figures S4 and S5 show that both the supporting electrolyte and pH affect the oxidation of diuron. In all conditions assessed, the diuron peak potential shifted to less positive values as pH increased, in agreement with prior observations for this analyte.^61^ The highest *I*_pa_ occurred at pH 3 in the BR buffer. Thus, the pH dependence aids peak discrimination and, therefore, analytical selectivity.

The decrease in *I*_pa_ signal at higher pH suggests that proton availability promotes the oxidation pathway in acidic media. We attribute this behavior to a proton-assisted oxidation pathway involving the amide/amine functionality of diuron, which promotes oxidative bond cleavage under acidic conditions, consistent with the scheme depicted in Figure 4 and prior reports.^62^ Based on these results, we selected the BR buffer at pH 3 for cyclic voltammetry (CV) and subsequent analytical determinations. Hence, the combined electrolyte and pH effects provide a practical lever to enhance the detectability and selectivity of diuron in complex matrices.

**Figure 4:**
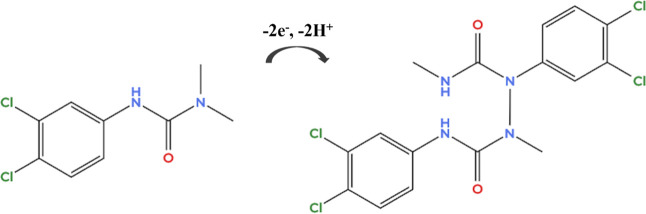
Proposed scheme for the electrochemical oxidation of diuron.

To investigate the influence of scan rate on diuron oxidation, we recorded cyclic voltammograms at pH 3 in BR buffer for CPS, Si_10_-CPS, Rice husk_10_-CPS, and MCM-41_10_-CPS over the 25–300 mV s^–1^ range (Figure 5a–d). For all electrodes, the anodic peak current (*I*_pa_) increased with scan rate, and plots of *I*_pa_ versus υ^1/2^ were linear (Figure 6, coefficients of determination in Table S2), indicating that diuron oxidation is governed by a diffusion-controlled process under these conditions.^61^

**Figure 5:**
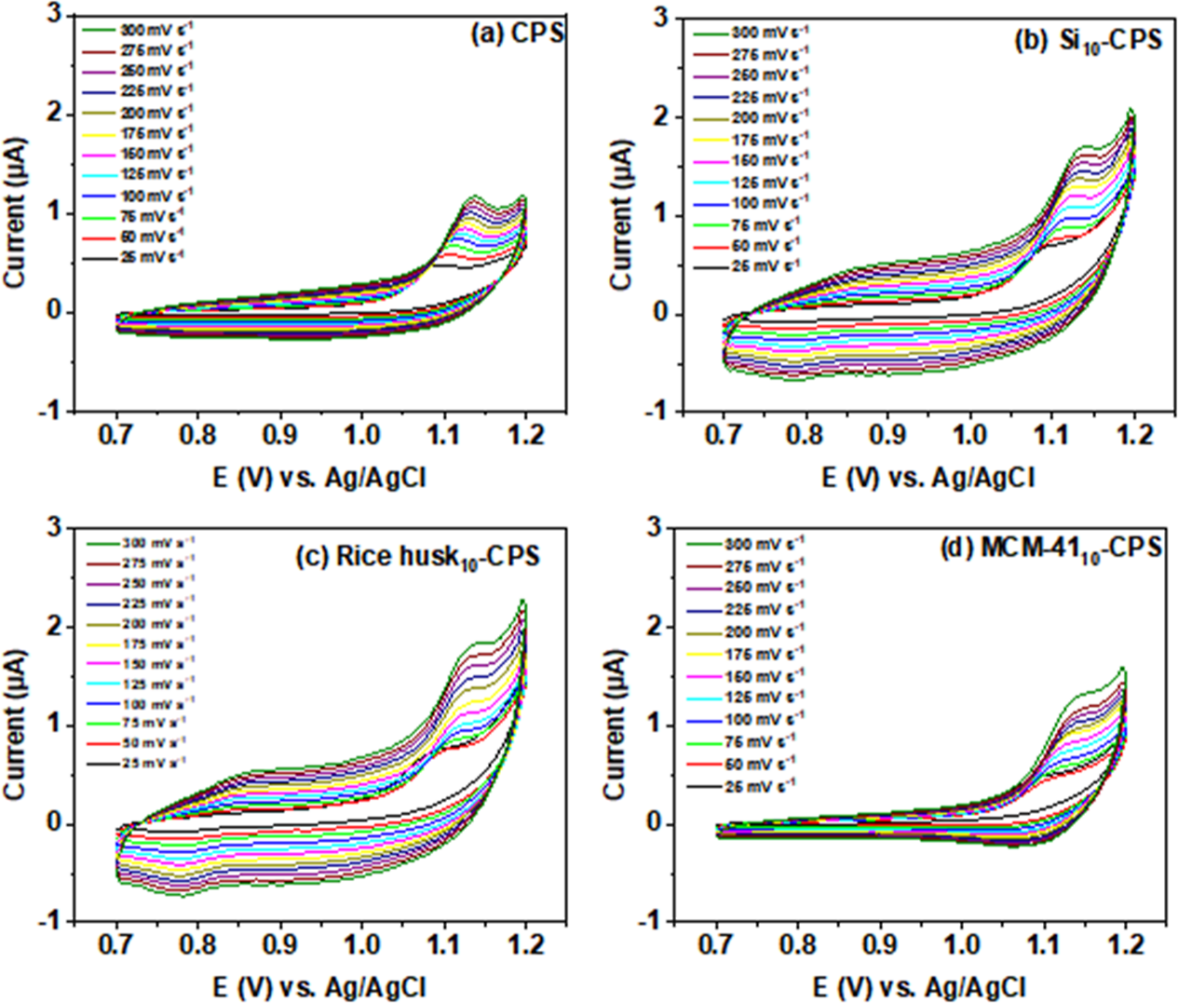
Cyclic voltammograms of (a) CPS, (b) Si_10_-CPS, (c) rice husk_10_-CPS, and (d) MCM-41_10_-CPS recorded in 0.1 mol L^–1^ BR buffer (pH 3.0) containing diuron at a fixed concentration, for scan rates between 25 and 300 mV s^–1^.

**Figure 6:**
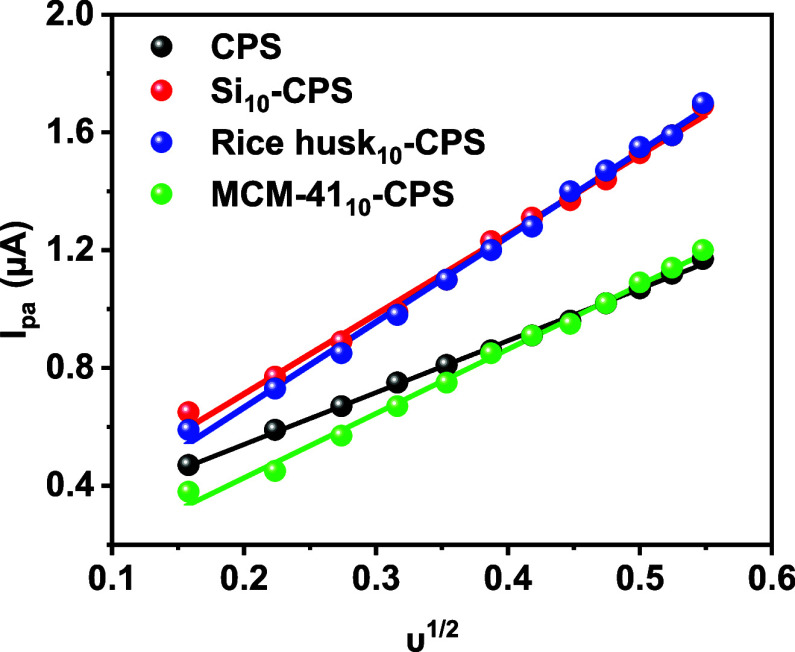
Dependence of the anodic peak current (*I*_pa_) observed in Figure 5 on the square root of the scan rate (υ^1/2^) for the four electrodes.

Initially, the voltammetric behavior in terms of intensity of *I*_pa_ for 2 mg L^–1^ diuron was compared using DPV and SWV at a scan rate of 20 mV s^–1^, a pulse time of 40 ms, and a pulse amplitude of 50 mV, as shown in Figure S6. DPV produced a cleaner voltammetric profile with a higher analytical signal than SWV under identical conditions. Relative to SWV (*n* = 3), the *I*_pa_ obtained by DPV was higher by 9.5 ± 0.2-fold for CPS, 4.3 ± 0.1-fold for Si_10_-CPS, 3.6 ± 0.1-fold for rice husk_10_-CPS, and 6.0 ± 0.3-fold for MCM-41_10_-CPS. This behavior is consistent with predominantly irreversible anodic oxidation, for which DPV typically provides superior resolution and sensitivity compared with SWV.^61^ Then, we selected DPV for subsequent quantification.

To obtain the best trade-off between analytical signal quality and peak current intensity, we optimized DPV parameters, pulse time (10–100 ms), pulse amplitude (20–100 mV), and scan rate (20–100 mV s^–1^), as shown in Figures S7–S9. The optimized conditions for further analytical measurements were a pulse time of 10 ms, a pulse amplitude of 100 mV, and a scan rate of 20 mV s^–1^, which maximized peak intensity while preserving reproducible, well-defined peaks.

#### 3.3.2 Calibration Curve

To quantify diuron, we constructed an external calibration by recording DPV responses in Britton–Robinson buffer at pH 3 over a concentration range of 0.002–2.0 mg L^–1^ diuron (Figure 7). The anodic peak current (*I*_pa_) increased with analyte concentration, and the calibration exhibited two linear dynamic ranges: 0.002–0.04 mg L^–1^ and 0.08–2.0 mg L^–1^. This behavior is consistent with the report by Morawski and co-workers, who also observed two distinct linear ranges for diuron detection using platinum/chitosan electrodes.^61^ Similarly, Sivaji and collaborators reported two linear regions (0.01–41.01 μM and 51.01–203.01 μM) in DPV-based diuron measurements.^63^

**Figure 7:**
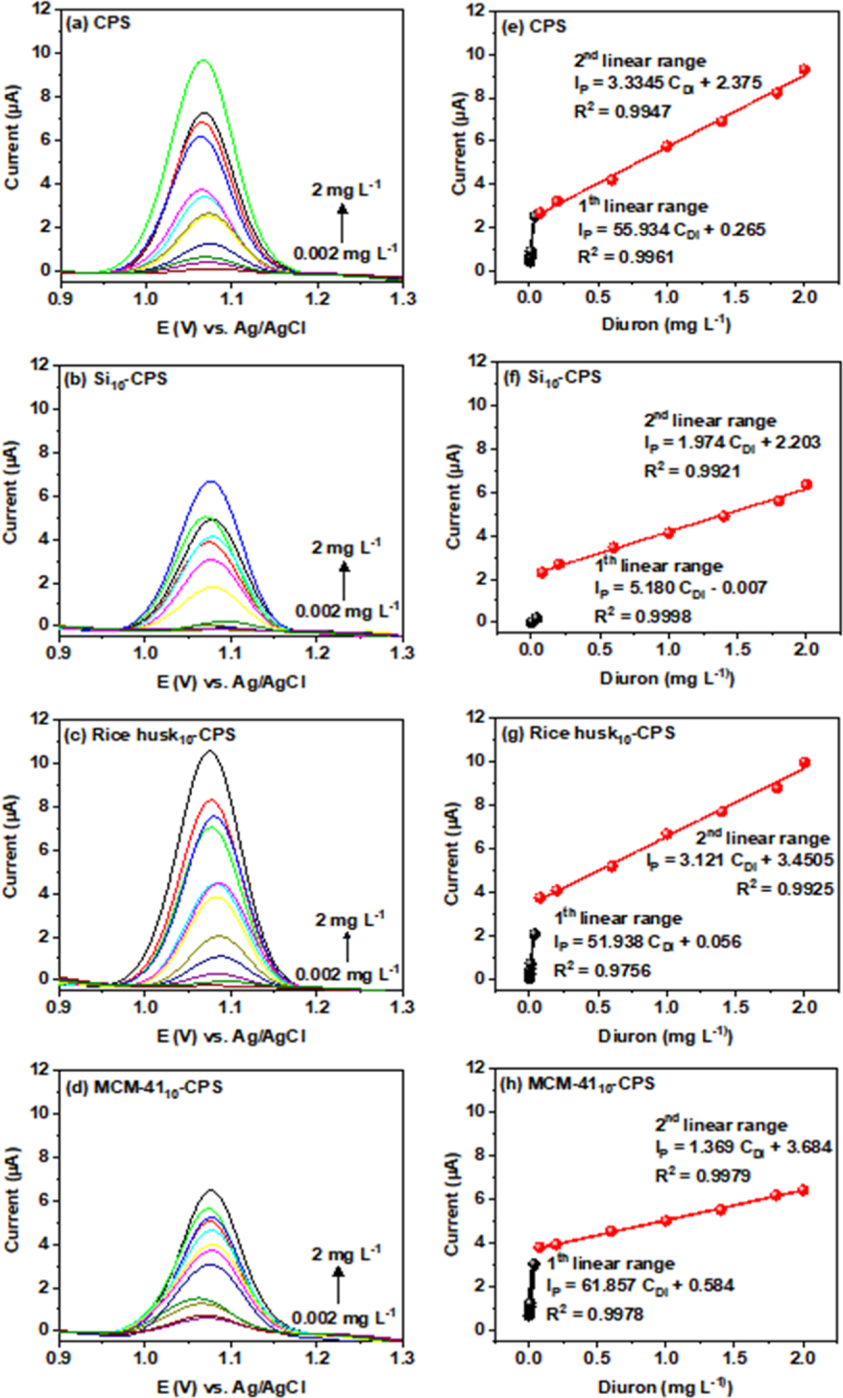
DPV calibration curves for diuron in BR buffer (pH 3): (a–d) voltammograms across 0.002 mg L^–1^–2.0 mg L^–1^ and (e–h) corresponding linear plots of *I*_pa_ versus concentration for the defined ranges.

The electrochemical performance of the Si_10_-CPS, rice husk_10_-CPS, and MCM-41_10_-CPS electrodes appears to be primarily influenced by pore accessibility and surface chemistry rather than by total surface area. Although MCM-41_10_-CPS exhibits an ordered mesoporous structure, partial pore blockage within the carbon paste electrode may reduce electrolyte penetration and hinder charge transfer. In contrast, Si_10_-CPS and rice husk_10_-CPS likely provide a more heterogeneous pore network and a higher density of surface silanol groups, which can enhance wettability and facilitate ionic transport, thereby contributing to a stronger electrochemical response. In general, Si_10_-CPS exhibits intermediate behavior relative to rice husk_10_-CPS and MCM-41_10_-CPS.

Regression analysis for both intervals showed strong correlations, highlighting the reliability of the modified electrodes for diuron determination under these conditions. We used the higher-concentration range (0.08–2.0 mg L^–1^) to estimate analytical figures of merit. The limits of detection (LOD) and quantification (LOQ) were calculated following the method described by Sawkar and co-workers,^51^ and the results are presented in Table 2.

**Table 2:** LOD and LOQ Calculated from the *I*_pa_ for CPS, Si_10_-CPS, Rice Husk_10_-CPS, and MCM-41_10_-CPS Measured by DPV in BR Buffer (pH 3) Over the 0.08–2.0 mg L^–1^ Range and in River Water Samples over the 0.25–2.0 mg L^–1^ Range

0.08*–*2.0 mg L^–1^	CPS	Si_10_-CPS	rice husk_10_-CPS	MCM-41_10_-CPS
LOD	2.13	3.34	3.31	8.07
LOQ	7.12	11.16	11.05	26.91

0.25*–*2.0 mg L^–1^	CPS	Si_10_-CPS	Rice husk_10_-CPS	MCM-41_10_-CPS
LOD	3.63	9.81	24.59	7.33
LOQ	12.10	32.70	81.98	24.43

The sensing platform demonstrated robust electroanalytical performance, supporting the use of silica-modified carbon-paste electrodes for diuron determination. We benchmarked the LOD calculated from *I*_pa_ against values reported in previous studies (Table 3). Although several reported systems achieve lower LODs, often by employing adsorptive preconcentration strategies or highly nanostructured catalytic interfaces, the present approach is designed for rapid, low-cost screening at mg L^–1^ levels. In this context, the proposed sensors are suitable for identifying contamination hotspots and selecting samples that require subsequent confirmatory analysis by more sensitive, resource-intensive methods.

**Table 3:** Comparison of LOD for Diuron Obtained with the Present Silica-Modified Carbon Paste Electrodes and with Representative Electrochemical Sensors Reported in the Literature^a^

sensor used	method	LOD	refs
CPS	DPV	2.13 mg L^–1^	this work
Si_10_-CPS	DPV	3.34 mg L^–1^	this work
rice husk_10_-CPS	DPV	3.31 mg L^–1^	this work
MCM-41_10_-CPS	DPV	8.07 mg L^–1^	this work
MWCNTs-CS@NGQDs/GCE	DPV	0.04 μg mL^–1^	^13^
PtNPs/CS/GCE	DPAdSV	20.00 μg L^–1^	^61^
MXene/PEI-MWCNTs	DPV	6.90 μg L^–1^	^64^
recycled ABS filaments for 3D printing	SWV	16.00 μg L^–1^	^65^
ZnFe_2_O_4_@CB	DPV	0.79 μg L^–1^	^63^
glassy carbon electrode	SWV	0.047 μg L^–1^	^66^
nanocrystalline cellulose modified carbon paste	SWV	81.55 μg L^–1^	^67^

a This work.

The developed sensors achieved screening-level limits of detection (LOD). Nonetheless, several advanced architectures, such as MWCNTs-CS@NGQDs/GCE and recycled ABS filaments for 3D printing, offer significantly lower detection limits (0.04 μg mL^–1^ and 16.00 μg L^–1^, respectively).

MWCNTs-CS@NGQDs/GCE: glassy carbon electrode modified with chitosan-encapsulated multiwalled carbon nanotubes combined with nitrogen-doped graphene quantum dots; PtNPs/CS/GCE: platinum/chitosan modified electrode; MXene/PEI-MWCNTs: highly conductive multilayer carbon nanotubes; ZnFe_2_O_4_@CB: zinc iron oxide@carbon black composite, and DPAdSV: differential pulse adsorptive stripping voltammetry.

In contrast, the rice husk_10_-CPS and MCM-41_10_-CPS electrodes emphasize simplicity, cost effectiveness, and a sustainable biosilica modifier, which are advantageous for practical environmental applications. These attributes underscore the value of biosilica as a functional additive that improves sensor performance while maintaining a clear sustainability focus. Typical drinking-water guideline values for diuron are around 0.02 mg L^–1^ in several jurisdictions (e.g., EU and Australia).^68,69^ Although the LOD obtained here (3.31 mg L^–1^) exceeds those thresholds, it positions the biosilica-based sensor as a sustainable, low-cost, and rapid screening tool for preliminary environmental assessment.

#### 3.3.3 Selectivity, Repeatability, and Reproducibility

The selectivity and signal stability of the developed sensors were evaluated by DPV at different time intervals. Selectivity was assessed in BR buffer (pH 3) at a fixed diuron concentration of 2 mg L^–1^ in the presence of potential coexisting species commonly found in river waters. Each interferent was tested at 100-fold excess relative to diuron, and changes in the *I*_pa_ and peak potential (*E*_p_) were used to quantify interference. The following species were examined: 4-nitrophenol, ametryn, carbaryl, acetaminophen, triclosan, trifularin, Cu^2+^, Fe^2+^, Hg^2+^, Pb^2+^, and a combined mixture of all interferents.

Figures S10–S13 compare DPV responses recorded in the absence and presence of each potential interferent. Only small variations in diuron *E*_p_ and *I*_pa_ were observed for all sensors, indicating that the analytical signal is largely preserved and that the electrodes exhibit good selectivity under the tested conditions. In CPS measurements, ametryn, triclosan, and trifluralin produced additional peaks centered near +0.80 V,^70,71^ while a lower-intensity peak at approximately +1.10 V, assigned to diuron, remained clearly discernible in the presence of ametryn and trifluralin. For all sensor configurations, acetaminophen showed an oxidation peak around +0.60 V,^72,73^ and signals attributed to Fe, Cu, and Hg appeared near +0.2 V.^74–76^ Nevertheless, none of these contributions caused significant changes in the diuron peak potential or intensity, further supporting the selectivity of the proposed sensors.

In measurements with the mixed interferent solution, all characteristic peaks were present; the diuron peak exhibited a slight positive shift and modest broadening but remained distinguishable. For comparison, Figures S11–S13 show the behavior of Si_10_-CPS, rice-husk_10_-CPS, and MCM-41_10_-CPS under identical interferent challenges. Notably, an additional peak at +0.52 V, consistent with carbaryl oxidation, was observed in Figures S11 and S12.^77^ These results indicate that the proposed sensors maintain adequate selectivity for diuron determination in the tested matrix.

Reusability and stability were examined via repeatability and reproducibility tests (Figure 8). Repeatability (intraday precision) was assessed with four consecutive measurements at 20 min intervals on a single day, first with surface renewal between runs (Figure 8a–d) and then without surface renewal (Figure 8e–h). With surface renewal, the oxidation peaks remained highly consistent for all electrodes, demonstrating excellent repeatability. Without renewal, some variation in peak height was observed; however, the electrodes retained sufficient sensitivity for diuron detection after multiple reuses. Across four reuse cycles, *I*_pa_ varied without a monotonic trend. The observed minimum–maximum ranges (in μA) were: CPS, 10.3–11.43, Si_10_-CPS, 5.75–7.96, Rice husk_10_-CPS, 7.44–12.09, and MCM-41_10_-CPS, 4.97–6.36. These intervals correspond to the lowest and highest peak currents recorded for each sensor over the four reuses.

**Figure 8:**
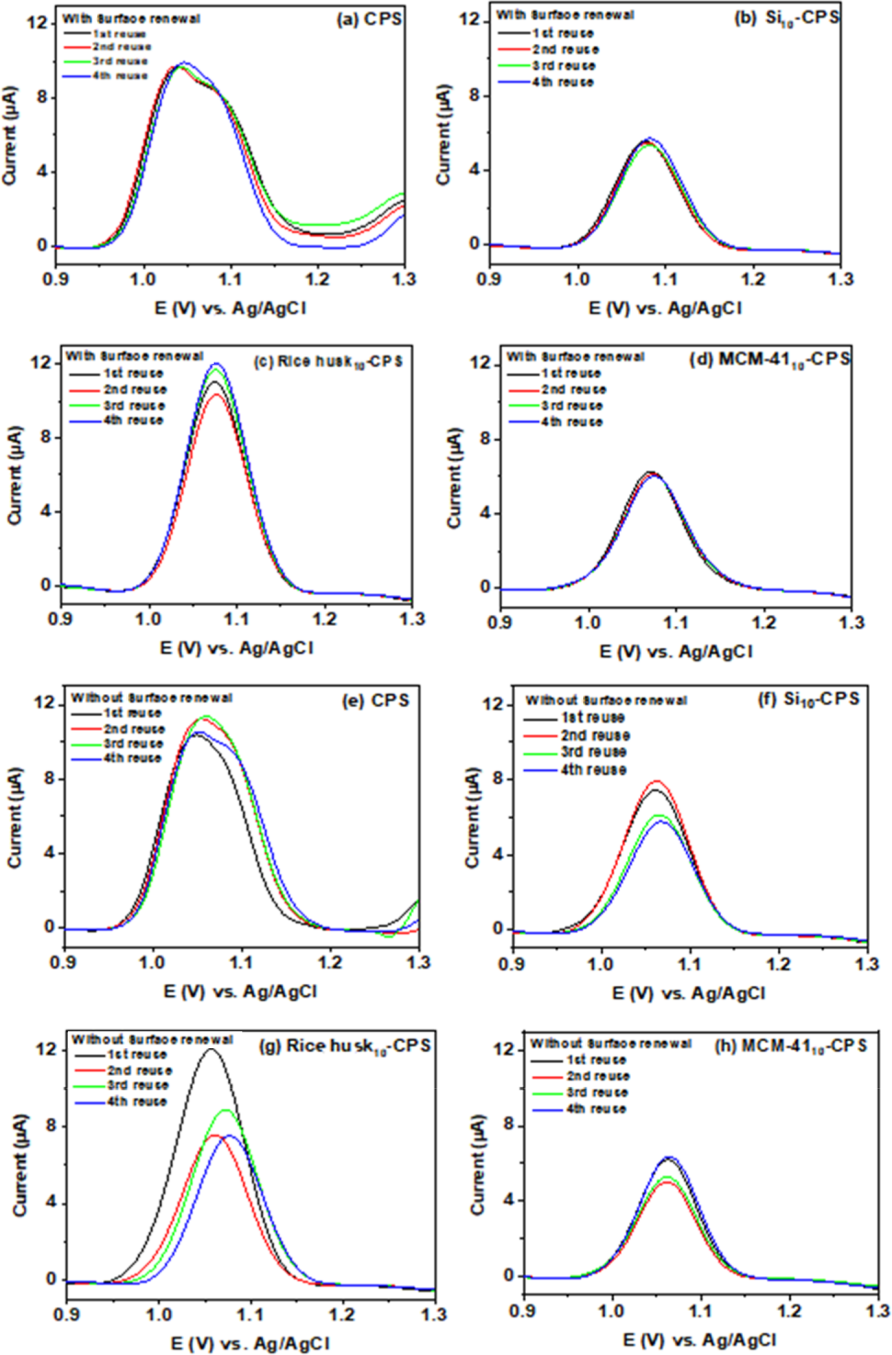
Repeatability analysis of CPS, Si_10_-CPS, rice-husk_10_-CPS, and MCM-41_10_-CPS for detecting 2 mg L^–1^ diuron in BR buffer (pH 3) by DPV: (a–d) repeatability with surface renewal between measurements; (e–h) repeatability analysis without surface renewal. The similar peak shapes and small variations in *I*_pa_ demonstrate satisfactory intraday repeatability and reusability for all silica-modified carbon-paste electrodes.

Reproducibility and storage stability were examined by storing the sensors at 2–4 °C for 30 days and re-evaluating their response (Figure 9). On day 0, *I*_pa_ values were 9.81 for CPS, 5.61 for Si_10_-CPS, 10.95 for rice husk_10_-CPS, and 6.25 for MCM-41_10_-CPS; after 30 days, the corresponding signals were 11.47, 6.52, 13.05, and 7.41, respectively, confirming stable or slightly enhanced responses over time. These findings demonstrate that the proposed silica-modified carbon-paste electrodes present satisfactory repeatability, reusability, and storage stability under the selected operating conditions.

**Figure 9:**
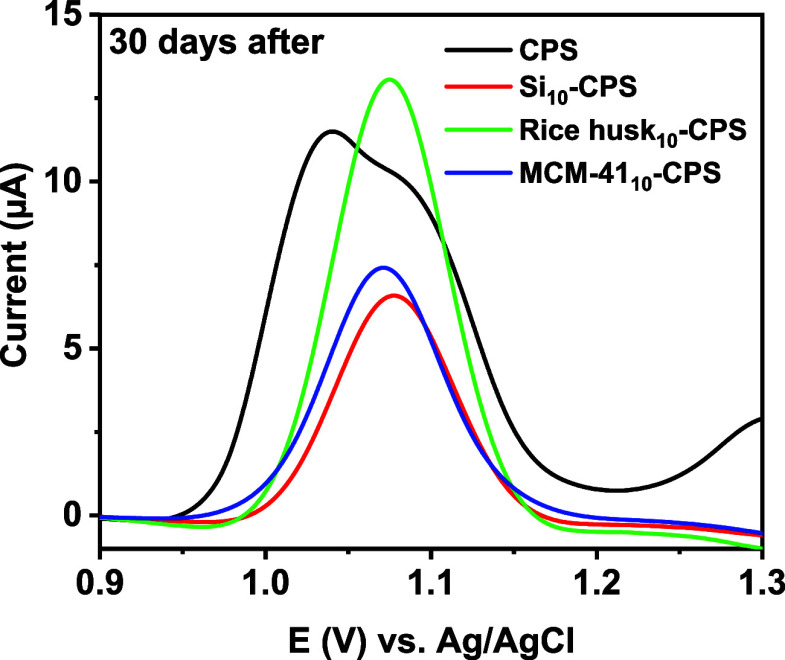
Reproducibility after 30 days of storage (postmanufacture) for CPS, Si_10_-CPS, rice-husk_10_-CPS, and MCM-41_10_-CPS in the detection of 2 mg L^–1^ diuron in BR buffer (pH 3) by DPV.

#### 3.3.4 Analysis of River Water Samples

CV was used to evaluate the electrochemical sensor responses in river water with and without added diuron. Figure 10 compares voltammograms recorded in the native matrix (blank river water) and after spiking with diuron at representative levels. In the absence of diuron, none of the electrodes exhibited well-defined faradaic peaks within the scanned potential window, indicating that the electrode surfaces did not catalyze intrinsic redox processes from the matrix under these conditions. Upon diuron addition, a broad anodic peak appeared at approximately +0.97 V for all sensors, consistent with the diuron oxidation observed in the buffer. These observations support the suitability of the proposed platform for diuron determination in river water, provided that calibration or standard addition accounts for matrix effects.

**Figure 10:**
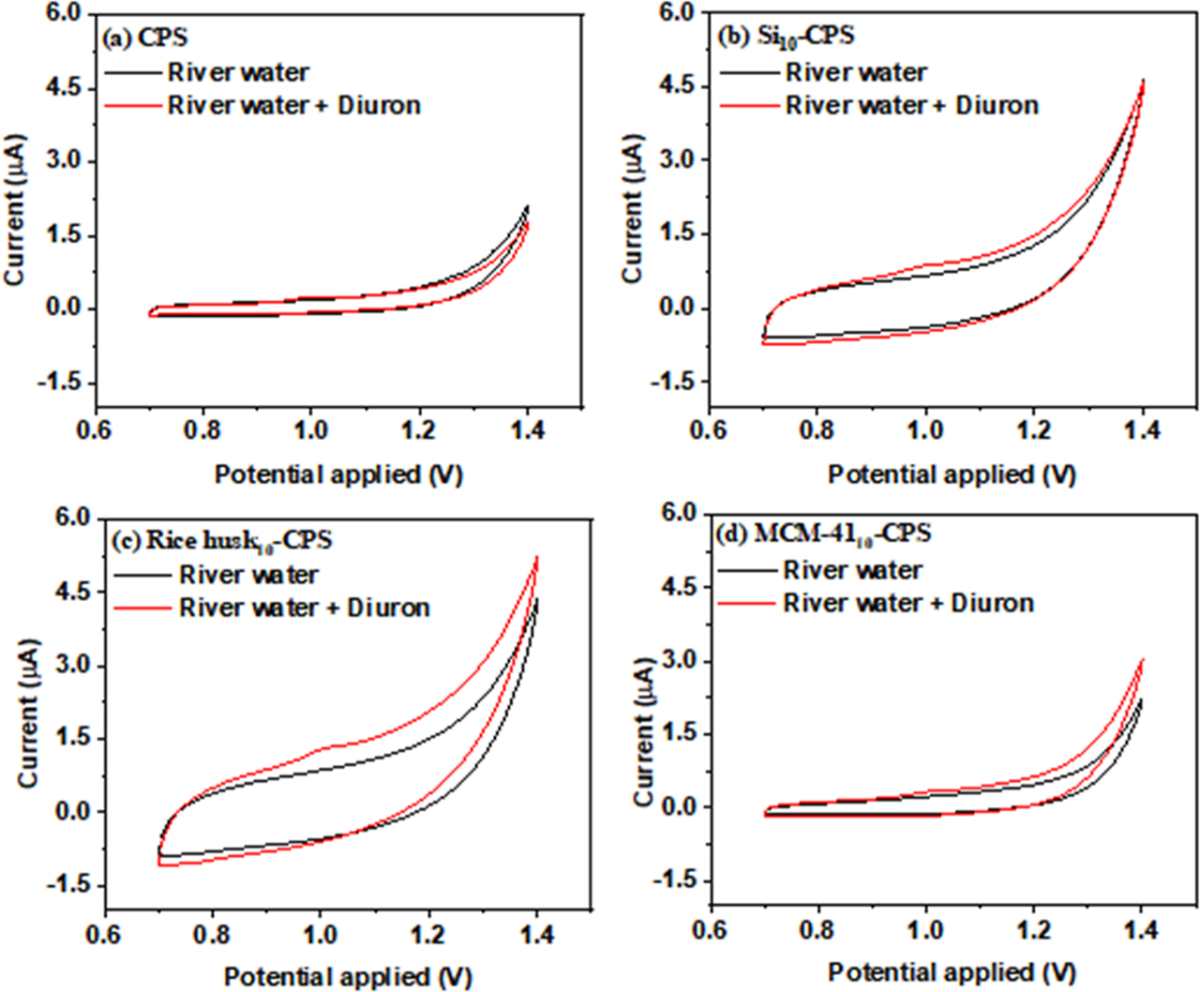
Cyclic voltammograms recorded in river water samples without and with 2 mg L^–1^ diuron using (a) CPS, (b) Si_10_-CPS, (c) rice husk_10_-CPS, and (d) MCM-41_10_-CPS electrodes at 20 mV s^–1^.

The lack of a well-defined diuron peak after spiking river water with 2.0 mg L^–1^ diuron at the CPS and MCM-41_10_-CPS electrodes (Figure 10a,d) is most plausibly associated with matrix effects intrinsic to river water. River water contains dissolved organic matter, inorganic ions, suspended colloids, and microbial components that can interfere with voltammetric detection by altering interfacial adsorption, blocking active sites, or increasing background currents. In particular, coadsorbing species may compete with diuron for electroactive sites on the electrode surface and suppress its faradaic response, thereby attenuating or obscuring the expected peak. In addition, differences in sample pH and buffering capacity relative to the supporting electrolyte can shift the acid–base speciation of diuron and modify its adsorption behavior and electron-transfer kinetics, further diminishing peak definition.

We validated quantification in river water using the standard addition method. Differential pulse voltammetry (DPV) measurements were performed on the native sample after successive additions of known diuron concentrations, and the resulting calibration (Figure 11) facilitated the accurate determination of diuron concentrations directly in the real matrix. This approach compensates for matrix effects by extrapolating the linear fit to the concentration axis; the intercept provides the endogenous diuron level, while the slope reflects sensitivity under matrix conditions. The standard-addition results support accurate quantification of diuron in river water using the proposed biosilica-modified electrodes.

**Figure 11:**
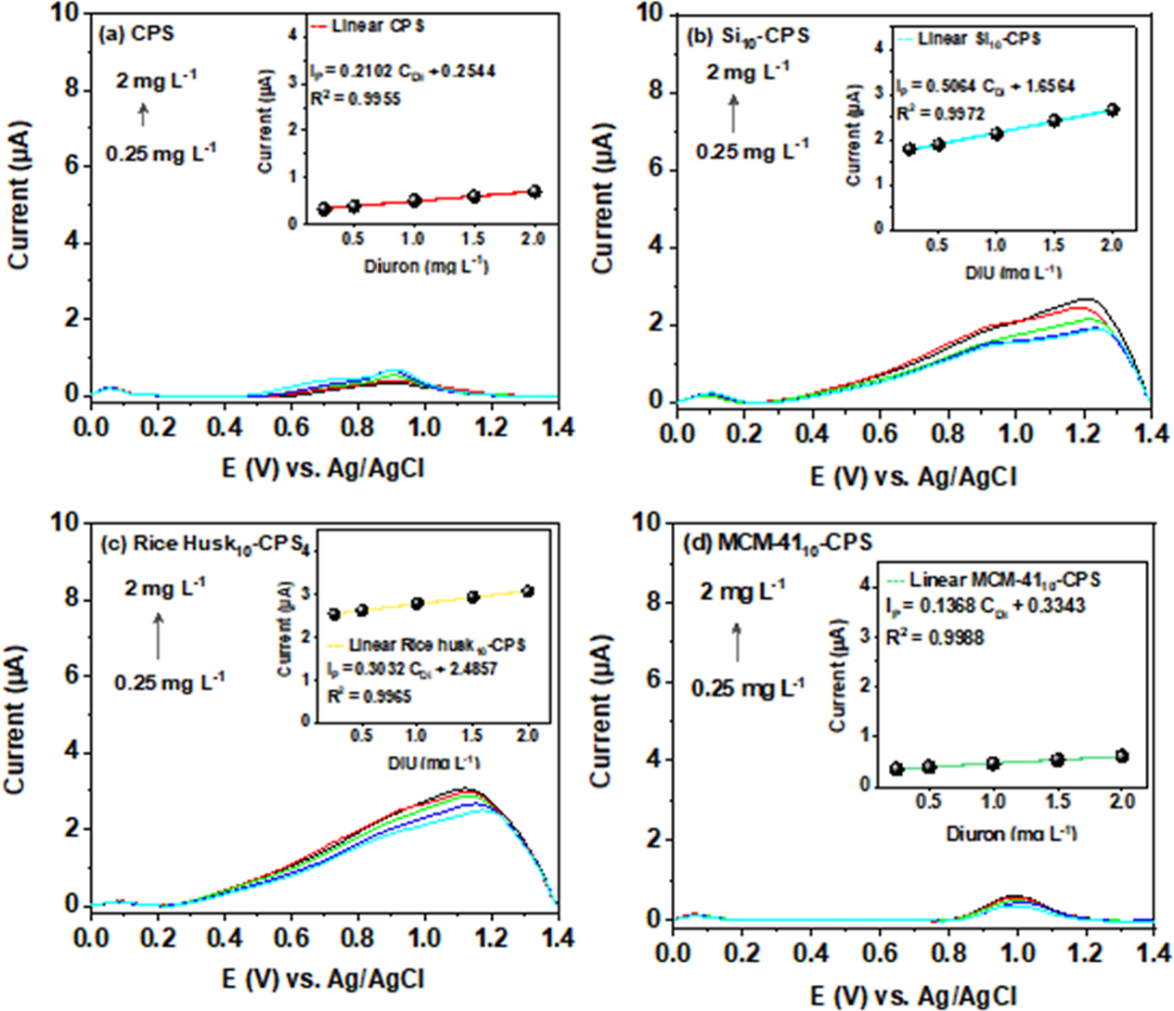
Standard-addition calibration curve of diuron (0.25–2.0 mg L^–1^) performed by DPV in river water for (a) CPS, (b) Si_10_-CPS, (c) rice husk_10_-CPS, and (d) MCM-41_10_-CPS electrodes. The inset shows the corresponding linear fits (*I*_pa_ versus added concentration) used to determine the native diuron content from the *x*-intercept.

A comparison of the LOD and LOQ values obtained in BR buffer and in river water (Table 2) highlights the expected influence of matrix effects on diuron sensing performance. For CPS, Si_10_-CPS, and rice husk_10_-CPS, the higher LOD/LOQ values in river water reflect the more complex composition of the natural matrix, including dissolved organic matter, inorganic ions, and coexisting pollutants that can compete for adsorption sites, modify double-layer properties, or promote mild surface fouling, thereby decreasing signal-to-noise ratios relative to the well-controlled buffer medium.^78^

In contrast, MCM-41_10_-CPS preserves comparable, or even slightly improved, LOD/LOQ values in river water compared with buffer, suggesting that the mesoporous silica framework provides a more accessible and selective microenvironment that mitigates matrix-induced losses in sensitivity. This behavior is consistent with reports that robust interfacial architectures or tailored micro/nanostructured modifiers are required to control matrix effects in real samples; many diuron sensors with very low nominal LODs achieve this only under ideal buffered conditions or by diluting, pre-extracting, or standard-addition-correcting environmental samples to suppress matrix interference.

Examples include rGO–AuNP^79^ or nanocomposite-based electrodes^80^ and deep-eutectic-solvent-modified platforms,^81^ which exhibit excellent LODs but often rely on sample dilution in BR buffer or relatively clean tap water to maintain performance. In this context, the ability of the silica-modified carbon-paste electrodes, particularly MCM-41_10_-CPS, to operate directly in river water with acceptable LOD/LOQ values and without extensive pretreatment underscores the practical robustness of the proposed biomass-derived modifiers for rapid screening in real environmental matrices.

Recovery experiments at three spike levels (0.30, 0.80, and 1.60 mg L^–1^) within the working range yielded satisfactory results for all sensors (Table 4). Recoveries fell within 90–110%, a range generally considered acceptable for quantitative analysis according to the Horwitz guidance.^61^ These results support the accuracy of the proposed electrochemical method for diuron in river water and indicate that matrix effects did not introduce significant bias under the tested conditions.

**Table 4:** Determination of Diuron in River Water Samples by DPV Using the Standard-Addition Method

sensor	diuron added (mg L^–1^)	diuron detected^a^ (mg L^–1^) ± SD^b^	% recovery
CPS	0.30	0.33 ± 0.01	110.00
	0.80	0.82 ± 0.03	102.50
	1.60	1.58 ± 0.04	98.75
Si_10_-CPS	0.30	0.29 ± 0.01	96.60
	0.80	0.78 ± 0.02	97.50
	1.60	1.62 ± 0.06	101.30
rice husk_10_-CPS	0.30	0.31 ± 0.02	103.30
	0.80	0.82 ± 0.01	102.50
	1.60	1.65 ± 0.03	103.10
MCM-41_10_-CPS	0.30	0.33 ± 0.03	110.00
	0.80	0.77 ± 0.04	96.30
	1.60	1.65 ± 0.08	103.10

a Average of three concurrent readings.

b Standard deviation.

## 4 Conclusions

This work demonstrates that biomass-derived silica can be used as an efficient electrochemical modifier to tailor charge-transfer properties and electroactive area of carbon paste electrodes, enabling robust voltammetric screening of diuron. Electron microscopy confirmed the formation of ordered, mesoporous MCM-41 using rice husk as the silica source, supporting a low-cost, sustainable platform. Electrochemical measurements (CV, DPV, and SWV) showed enhanced responses for diuron relative to unmodified CPS, consistent with increased electroactive area and facilitated interfacial charge transfer. Complementary EIS indicated lower charge-transfer resistance for the biosilica-modified electrodes, aligning with the improved voltammetric currents. Analytically, DPV provided the most suitable readout; optimization yielded well-defined peaks, two linear dynamic ranges, and screening-level LODs in buffer. The sensors exhibited adequate selectivity against common cocontaminants and acceptable precision and recovery in river water using standard addition, supporting applicability to environmental samples. Thus, this work advances sustainable materials valorization for electrochemical sensing and highlights rice-husk biosilica as a viable modifier for rapid environmental monitoring of phenylurea herbicides. Future efforts should target preconcentration strategies and device engineering to lower detection limits in complex matrices and enable on-site deployment.

## Supplementary Material



## Notes

Additional electrochemical characterization and optimization data for CPS, SiO_2_-, rice-husk biosilica-, and MCM-41-modified carbon paste sensors, including cyclic voltammograms and scan-rate dependence in 1.0 mmol L^–1^ [Fe­(CN)_6_]^3–/4–^ (Figures S1 and S2; Table S1), DPV responses in different buffer media and pH conditions (Figures S3–S5; Table S2), and optimization of SWV/DPV parameters as a function of pulse time, amplitude, and scan rate (Figures S6–S9). Selectivity studies in BR buffer (pH 3) for all electrodes in the presence of common organic and inorganic interferents (4-nitrophenol, acetaminophen, ametryn, carbaryl, triclosan, trifluralin, Cu^2+^, Fe^2+^, Hg^2+^, Pb^2+^, and their mixture) are also provided (Figures S10–S13) (PDF)
