# Supplementary material for: Sustainable Voltammetric Sensors Based on Rice-Husk-Derived Biosilica for Rapid, Low-Cost, and Selective Determination of Diuron in Water
Source: ACS Omega. 2026 Feb 15;11(7):12622–36. doi: 10.1021/acsomega.5c12781 (PMC12947228; doi:10.1021/acsomega.5c12781)
Supplement: Supplementary file 1 [file ao5c12781_si_001.pdf]

Supplementary information for the paper

Sustainable voltammetric sensors based on rice-husk-derived biosilica for rapid, low-cost, and selective determination of diuron in water

Roberta A. de Jesus[a], *Gustavo V. de S. Santos*[b,c], *José A. do S. Costa*[d], *Katlin I. B. Eguiluz*[b,c], *Giancarlo R. Salazar-Banda*[b,c,*], *and Zaine T. Camargo*[a,*]

[a] Department of Chemistry, Federal University of Sergipe, 49100-000, São Cristóvão, Sergipe, Brazil

[b] Laboratory of Electrochemistry and Nanotechnology, Institute of Technology and Research (ITP), 49032-490, Aracaju, Sergipe, Brazil

[c] Process Engineering Graduate Program (PEP), Tiradentes University, 49032-490, Aracaju, Sergipe, Brazil

[d] Study Group on Structured Nanomaterials, Federal University of Western Pará, 68040070, Santarém, Pará, Brazil

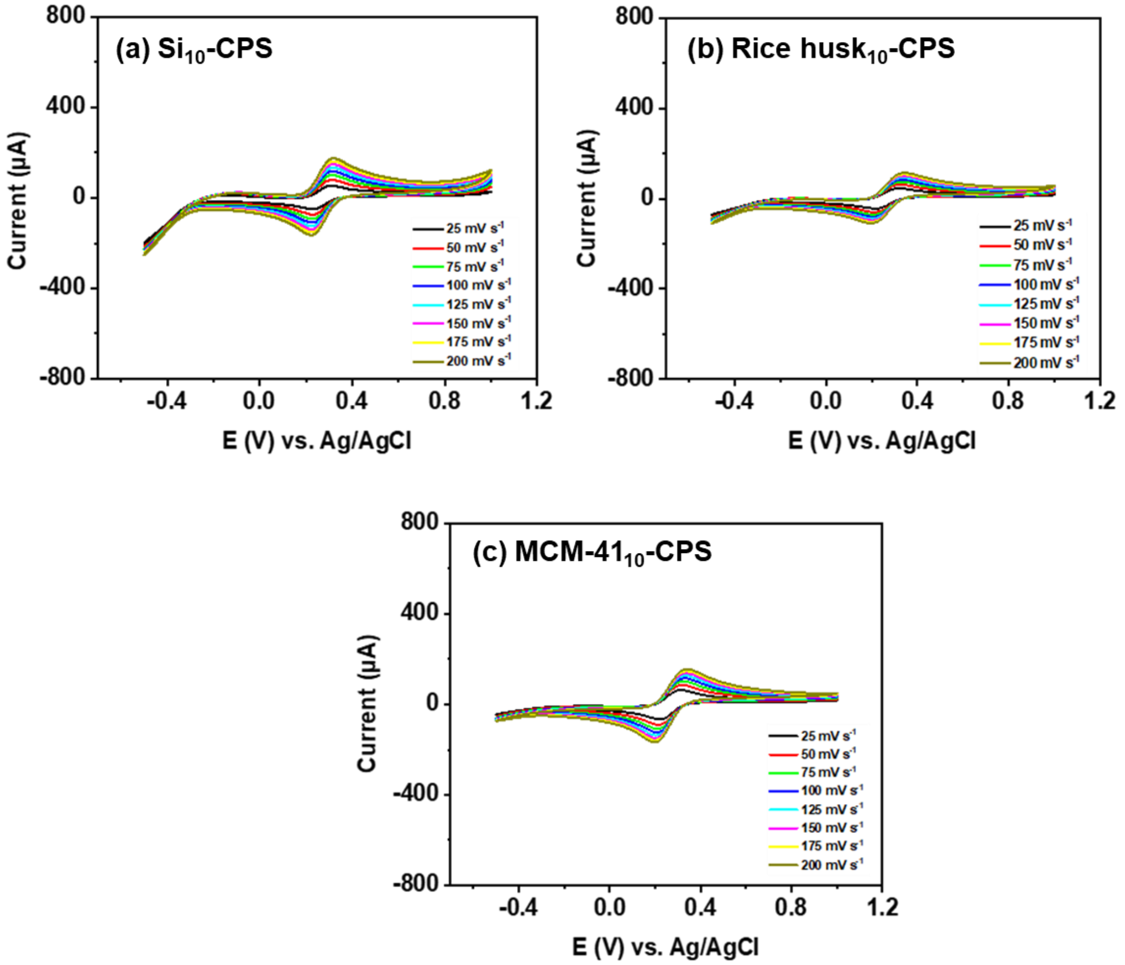

Figure S1. Cyclic voltammograms recorded at different scan rates (25–200 mV s^{-1}) for (a) Si_{10}-CPS, (b) Rice $husk_{10}$-CPS, and (c) MCM-41_{10}-CPS electrodes in 1.0 mmol L^{-1} $[Fe(CN)_6]^{3-}/[Fe(CN)_6]^{4-}$solution.

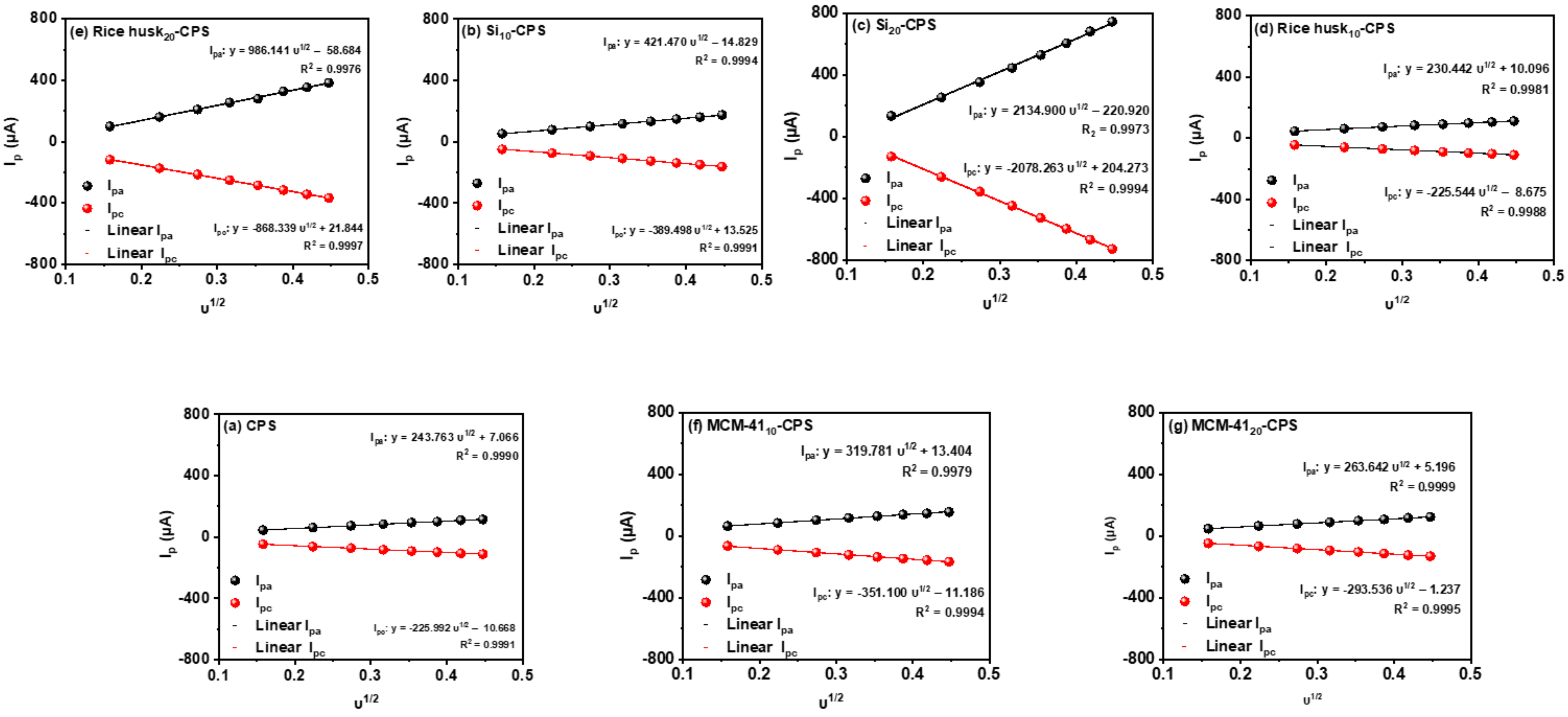

Figure S2. The regression plots obtained at different scan rates (25–200 mV s^{-1}) for the (a) CPS, (b) Si_{10}-CPS, (c) Si_{20}-CPS, (d) Rice $husk_{10}$-CPS, (e) Rice $husk_{20}$-CPS, and (f) MCM-41_{10}-CPS and (g) MCM-41_{20}-CPS electrodes in 1 mmol L^{-1} $[Fe(CN)_6]^{3-/4-}$ solution.

Table S1. Calculated values of I_{pa} /I_{pc} and ΔEp for CPS, Si_{10}-CPS, Si_{20}-CPS, Rice $husk_{10}$-CPS, Rice $husk_{20}$-CPS, MCM-41_{10}-CPS, and MCM-41_{20}-CPS

		**CPS**		**Si_{10}-CPS**		**Si_{20}-CPS**		**Rice $husk_{10}$-CPS**		**Rice $husk_{20}$-CPS**		**$MCM41_{10}$-CPS**		**MCM-41_{20}-CPS**	
Scan rate ($mV\ s^{-1}$)	$\upsilon^{1/2}$ ($mV\ s^{-1}$)	ΔEp (mV/n)	I_{pa}/I_{pc}	ΔEp (mV/n)	I_{pa}/I_{pc}	ΔEp (mV/n)	I_{pa}/I_{pc}	ΔEp (mV/n)	I_{pa}/I_{pc}	ΔEp (mV/n)	I_{pa}/I_{pc}	ΔEp (mV/n)	I_{pa}/I_{pc}	ΔEp (mV/n)	I_{pa}/I_{pc}
**25**	5.0	120	0.98	72	1.08	60	1.02	104	1.03	130	0.85	102	0.97	85	0.99
**50**	7.1	144	1.00	81	1.07	81	0.97	117	1.05	145	0.93	104	0.92	88	0.96
**75**	8.7	141	1.03	88	1.09	92	0.98	123	1.05	155	0.98	117	0.94	91	0.95
**100**	10.0	154	1.08	89	1.09	97	0.99	133	1.05	152	1.02	122	0.94	95	0.94
**125**	11.2	159	1.03	89	1.06	102	1.00	135	1.03	160	0.86	130	0.94	97	0.95
**150**	12.2	157	1.03	98	1.09	106	1.01	144	1.03	172	1.04	134	0.94	97	0.93
**175**	13.2	167	0.95	95	1.08	110	1.02	140	1.04	176	1.03	140	0.93	100	0.93
**200**	14.1	169	1.04	97	1.08	115	1.02	150	1.03	186	1.05	140	0.92	105	0.93

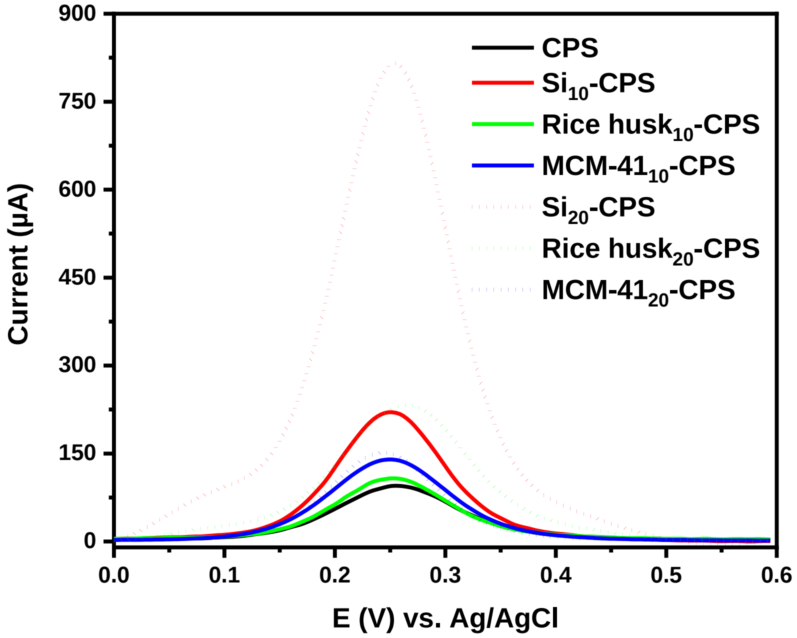

Figure S3. DPV of 1.0 mmol L^{-1} $[Fe(CN)6]^{3-/4-}$ in 1.0 mol L^{-1} KCl, recorded using CPS, Si_{10}-CPS, Si_{20}-CPS, Rice $husk_{10}$-CPS, Rice $husk_{20}$-CPS, MCM-41_{10}-CPS, and MCM-41_{20}-CPS at 20 mV s^{-1}, pulse time 40 ms, and amplitude 50 mV.

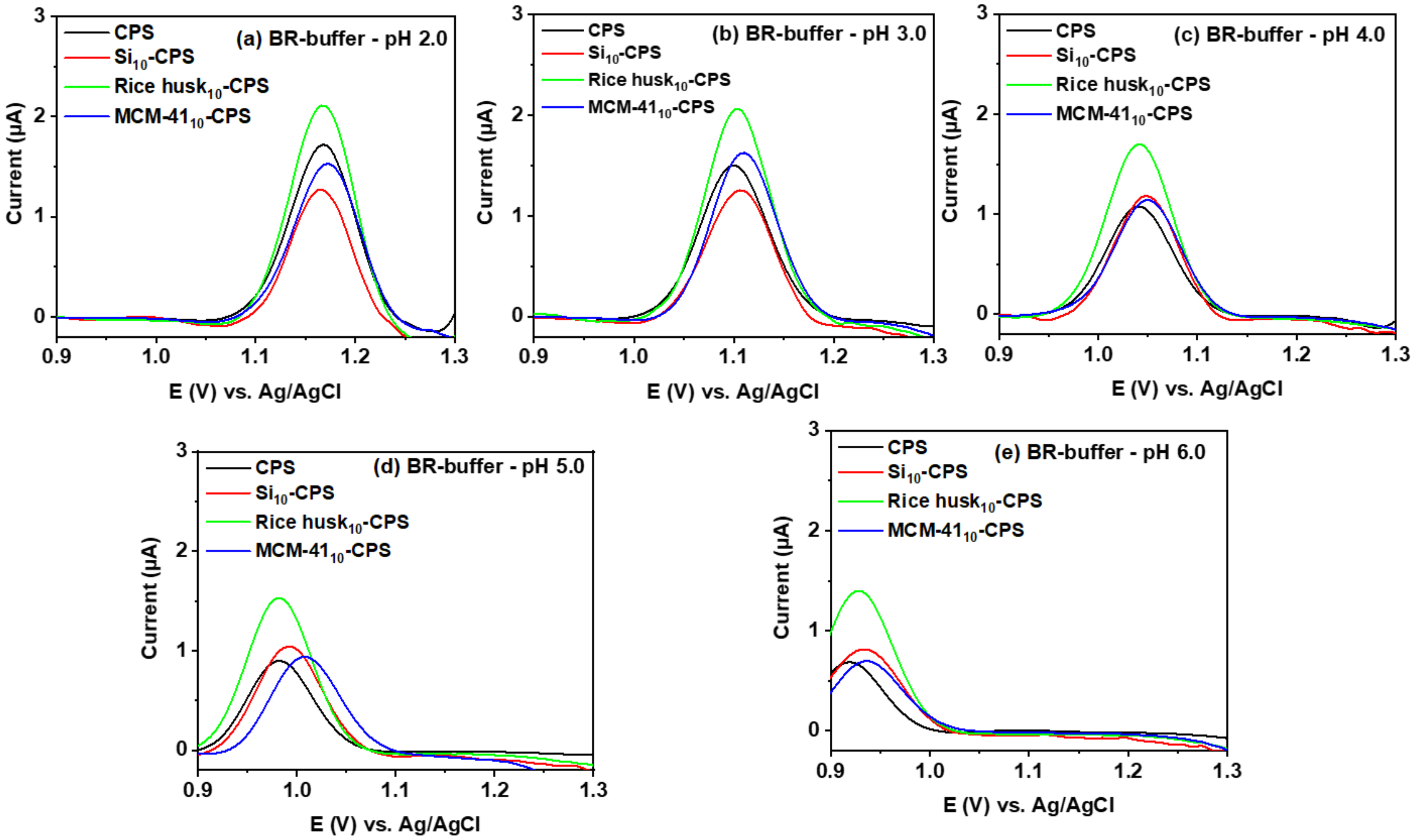

Figure S4 – DPV responses of the electrodes recorded in BR buffer solution containing Diuron (2 mg L^{-1}) at different pH values: (a) pH 2, (b) pH 3, (c) pH 4, (d) pH 5, and (e) pH 6.

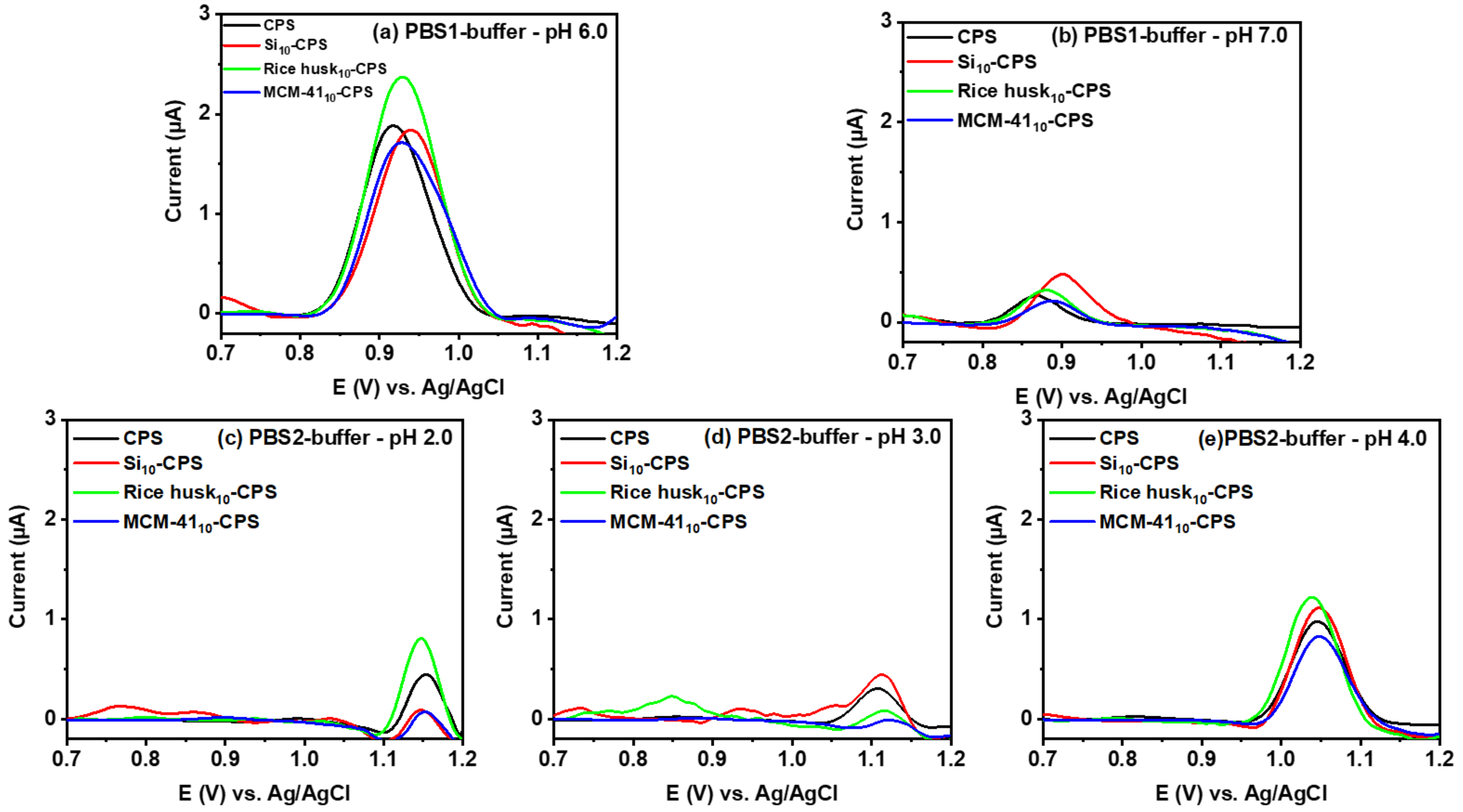

Figure S5. DPV responses recorded for each electrode in phosphate buffer solution 1 (PBS1) at (a) pH 6 and (b) pH 7, and in phosphate buffer solution 2 (PBS2) at (c) pH 2, (d) p H3, and (e) pH 4, in the presence of diuron (2 mg L^{-1}).

Table S2. Linear regressions of I_{pa} versus $\upsilon^{1/2}$ in BR pH 3

**Electrode**	**Regression parameters**
CPS	$\mathbf{I_{pa}}$**:** $y = 1.309\ \upsilon^{1/2} - 0.0359$<br>$R^2 = 0.9949$
Si_{10}-CPS	$\mathbf{I_{pa}}$**:** $y = 5.188\ \upsilon^{1/2} + 0.718$<br>$R^2 = 0.9867$
Rice $husk_{10}$-CPS	$\mathbf{I_{pa}}$**:** $y = 13.469\ \upsilon^{1/2} + 0.721$<br>$R^2 = 0.9880$
MCM-41_{10}-CPS	$\mathbf{I_{pa}}$**:** $y = 1.575\ \upsilon^{1/2} - 0.053$<br>$R^2 = 0.9961$

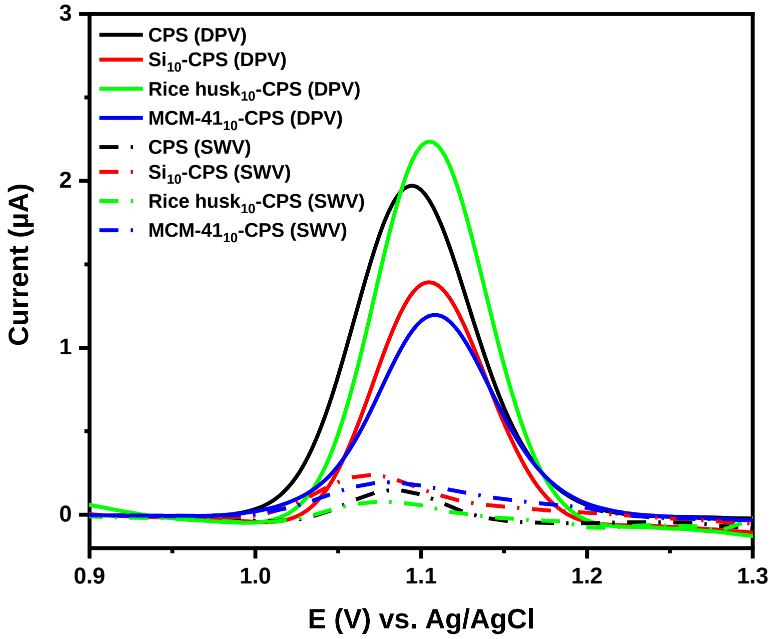

Figure S6. SWV and DPV optimization studies recorded in BR buffer (pH 3) in the presence of diuron (2 mg L^{-1}).

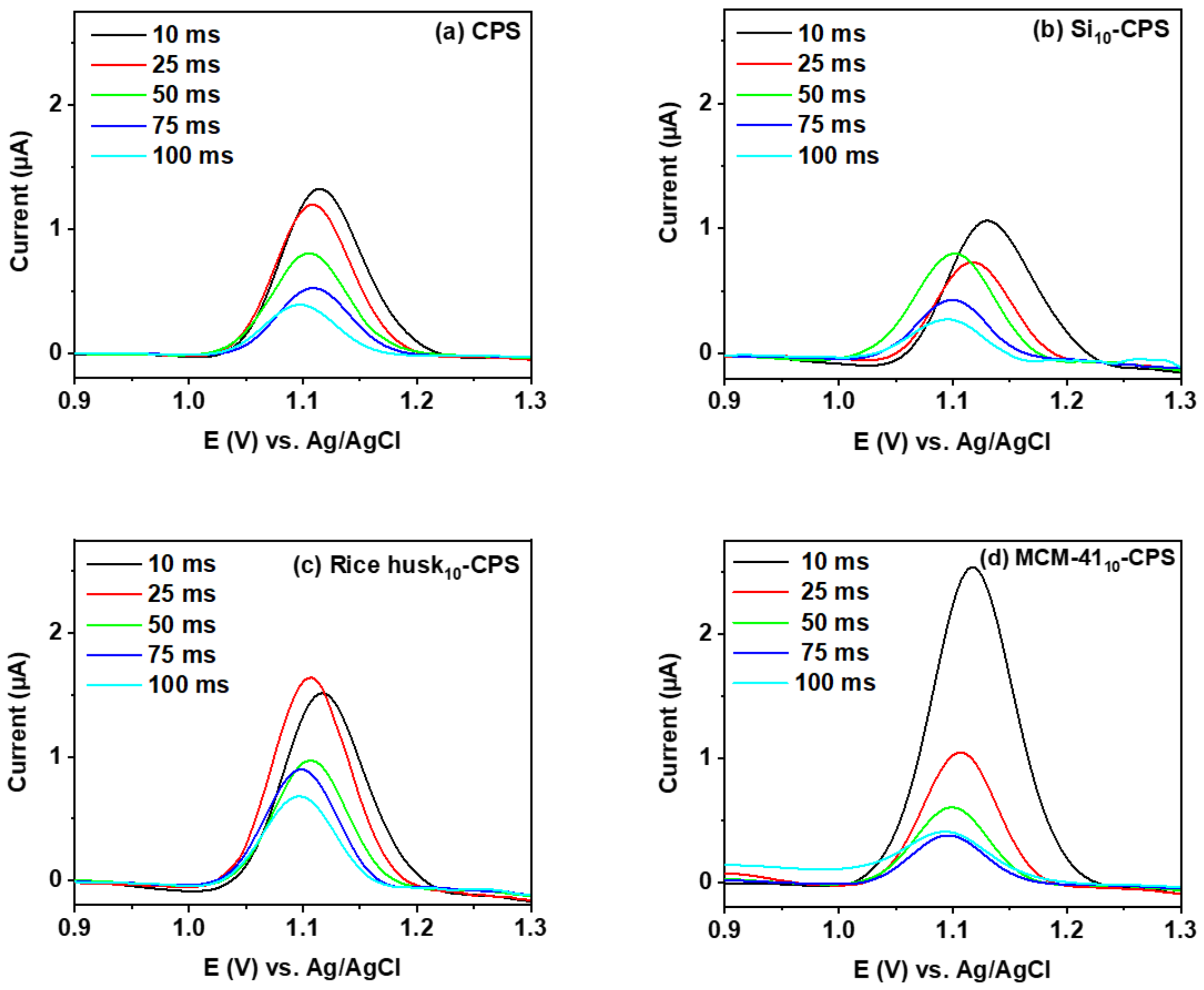

Figure S7. DPV optimization studies for (a) CPS, (b) Si_{10}-CPS, (c) Rice $husk_{10}$-CPS, and (d) MCM-41_{10}-CPS recorded in BR buffer (pH 3) containing diuron (2 mg L^{-1}) as a function of pulse time (10 to 100 ms).

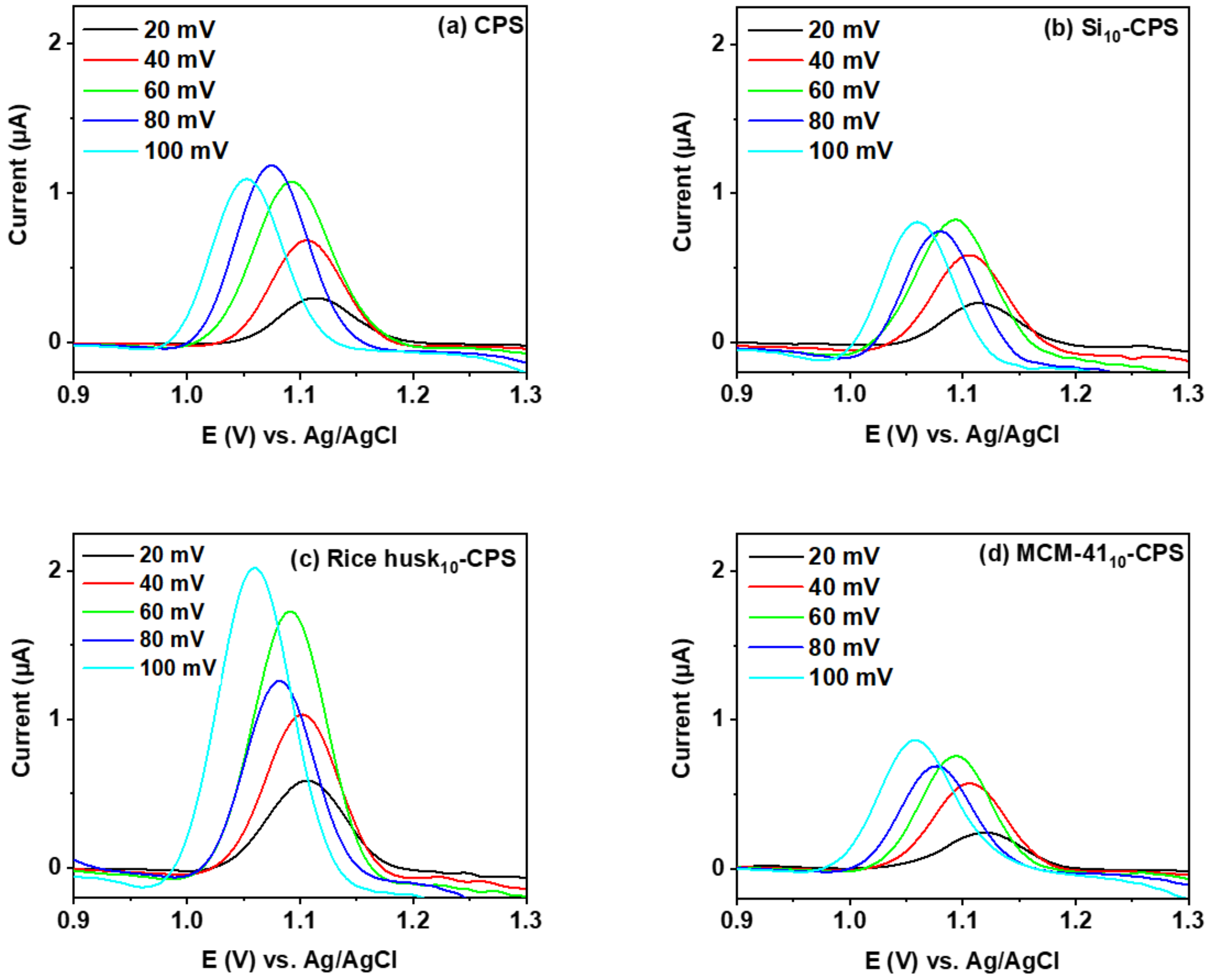

Figure S8. DPV optimization studies for (a) CPS, (b) Si_{10}-CPS, (c) Rice $husk_{10}$-CPS, and (d) MCM-41_{10}-CPS recorded in BR buffer (pH 3) containing diuron (2 mg L^{-1}), as a function of pulse amplitude (20 to 100 mV).

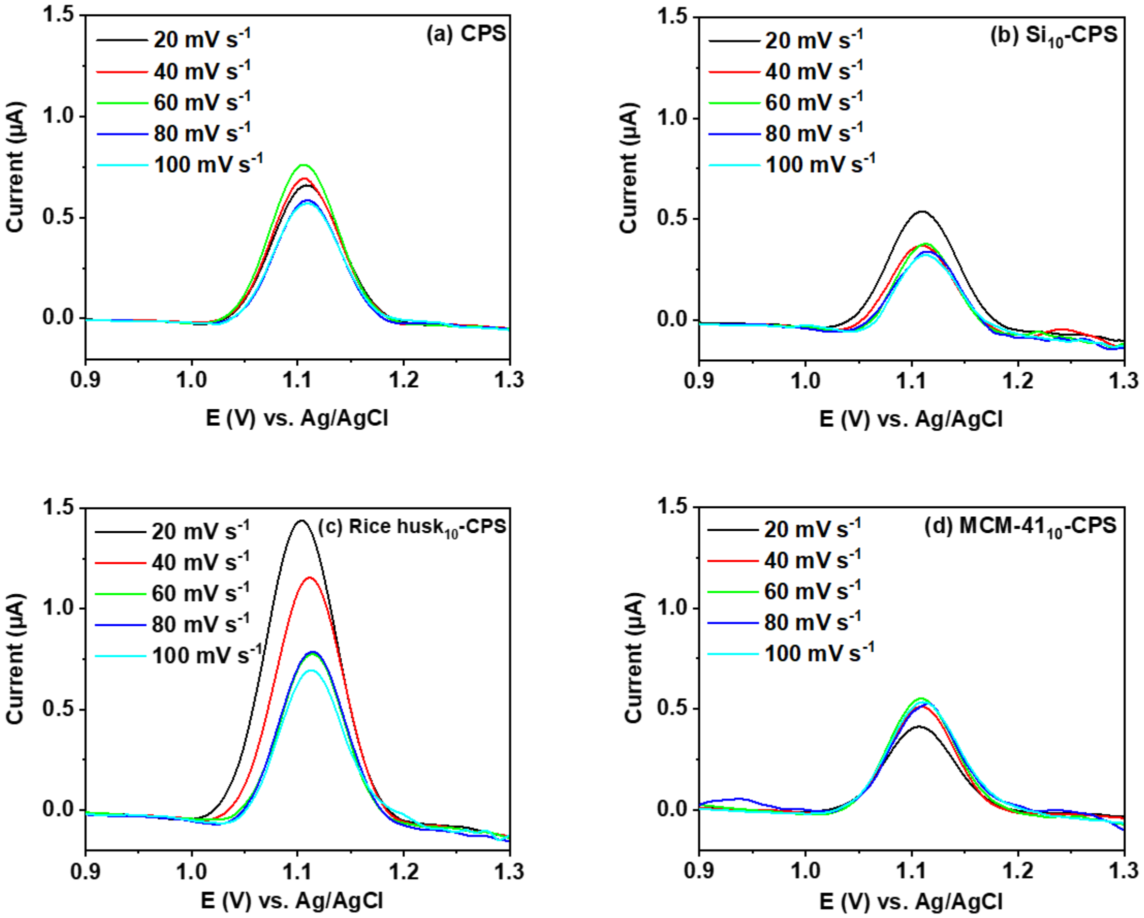

Figure S9. DPV optimization studies for (a) CPS, (b) Si_{10}-CPS, (c) Rice $husk_{10}$-CPS, and (d) MCM-41_{10}-CPS recorded in BR buffer (pH 3) containing diuron (2 mg L^{-1}), as a function of scan rate (20 to 100 mV s^{-1}).

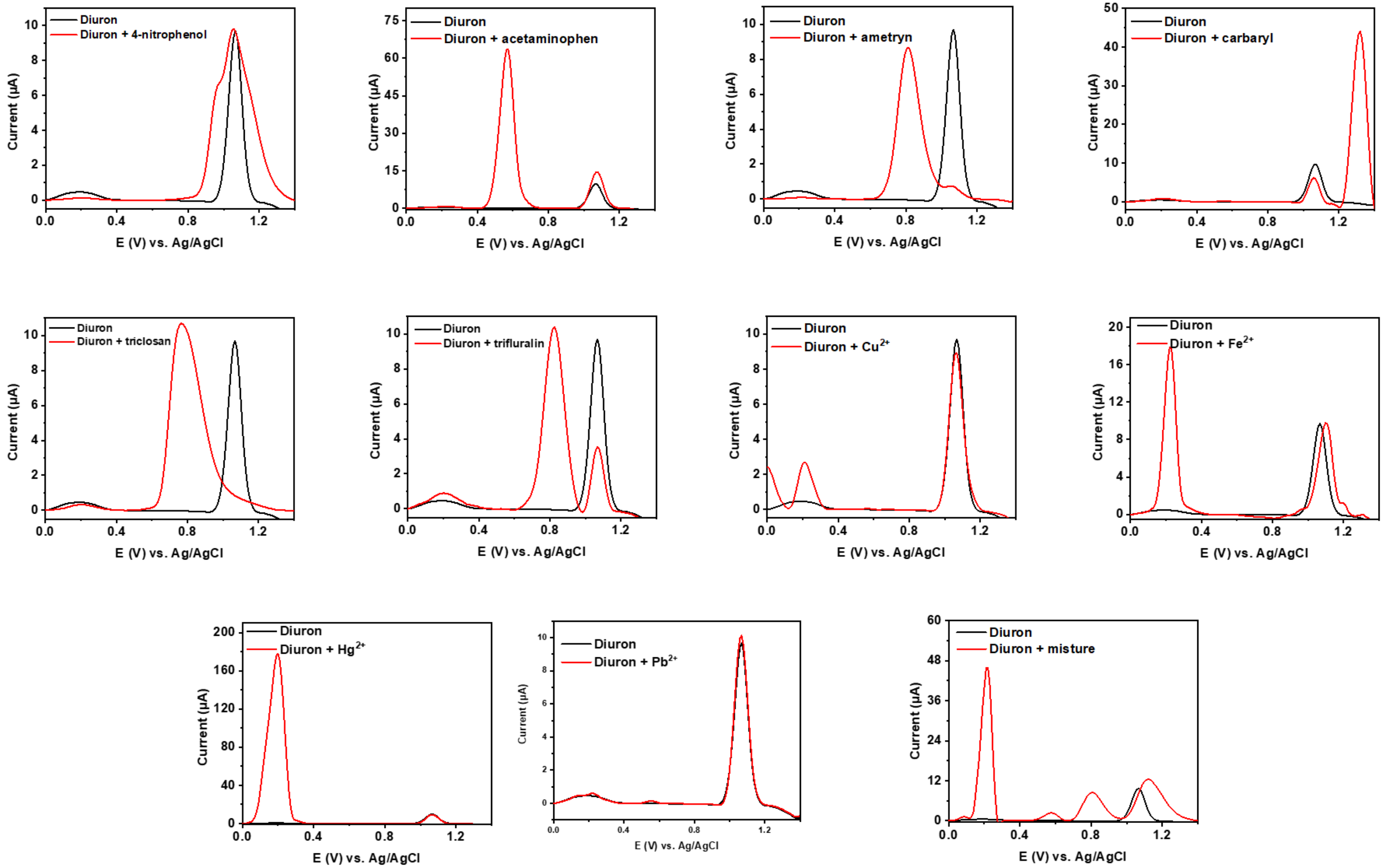

Figure S10. Selectivity study for CPS in BR buffer (pH 3) with diuron (2 mg L^{-1}) in the presence of the following interferents: 4-nitrophenol, acetaminophen, ametryn, carbaryl, triclosan, trifluralin, Cu^{2+}, Fe^{2+}, Hg^{2+}, Pb^{2+}, and their mixture.

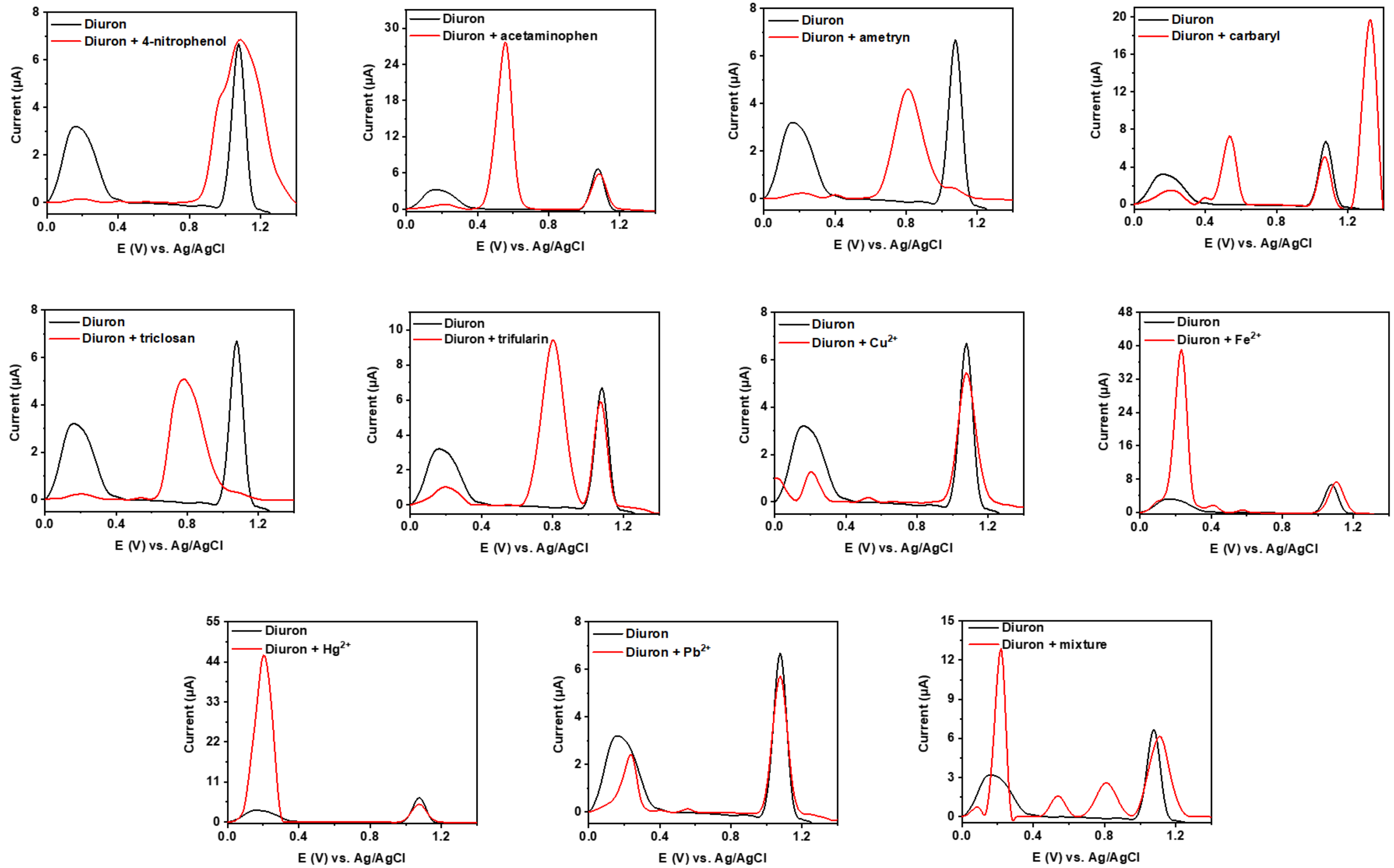

Figure S11. Selectivity study for Si_{10}-CPS in BR buffer (pH 3) with diuron (2 mg L^{-1}) in the presence of the following interferents: 4-nitrophenol, acetaminophen, ametryn, carbaryl, triclosan, trifluralin, Cu^{2+}, Fe^{2+}, Hg^{2+}, Pb^{2+}, and their mixture.

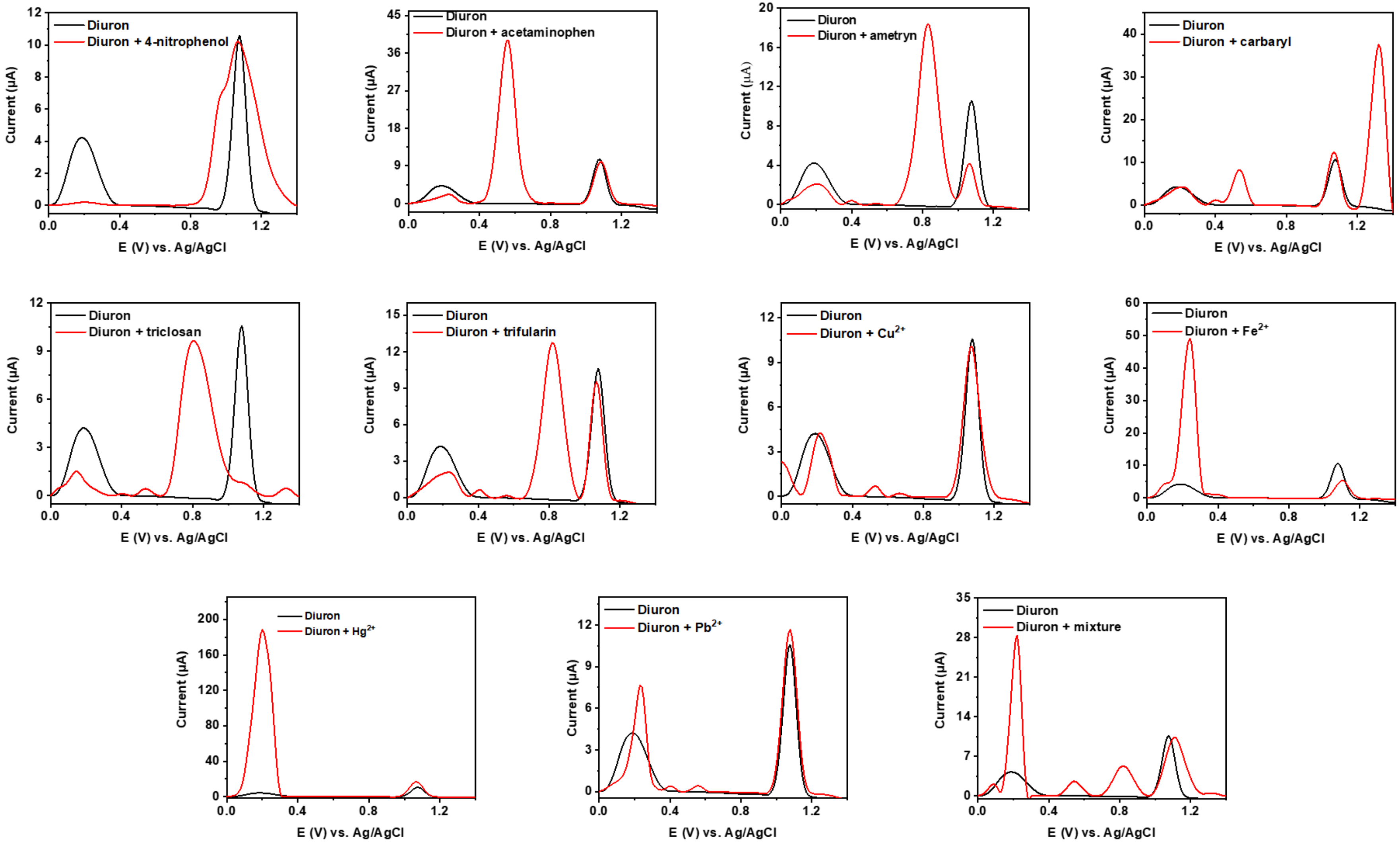

Figure S12. Selectivity study for Rice husk$_{10}$-CPS in BR buffer (pH 3) with diuron (2 mg L^{-1}) in the presence of the following interferents: 4-nitrophenol, acetaminophen, ametryn, carbaryl, triclosan, trifluralin, Cu^{2+}, Fe^{2+}, Hg^{2+}, Pb^{2+}, and their mixture.

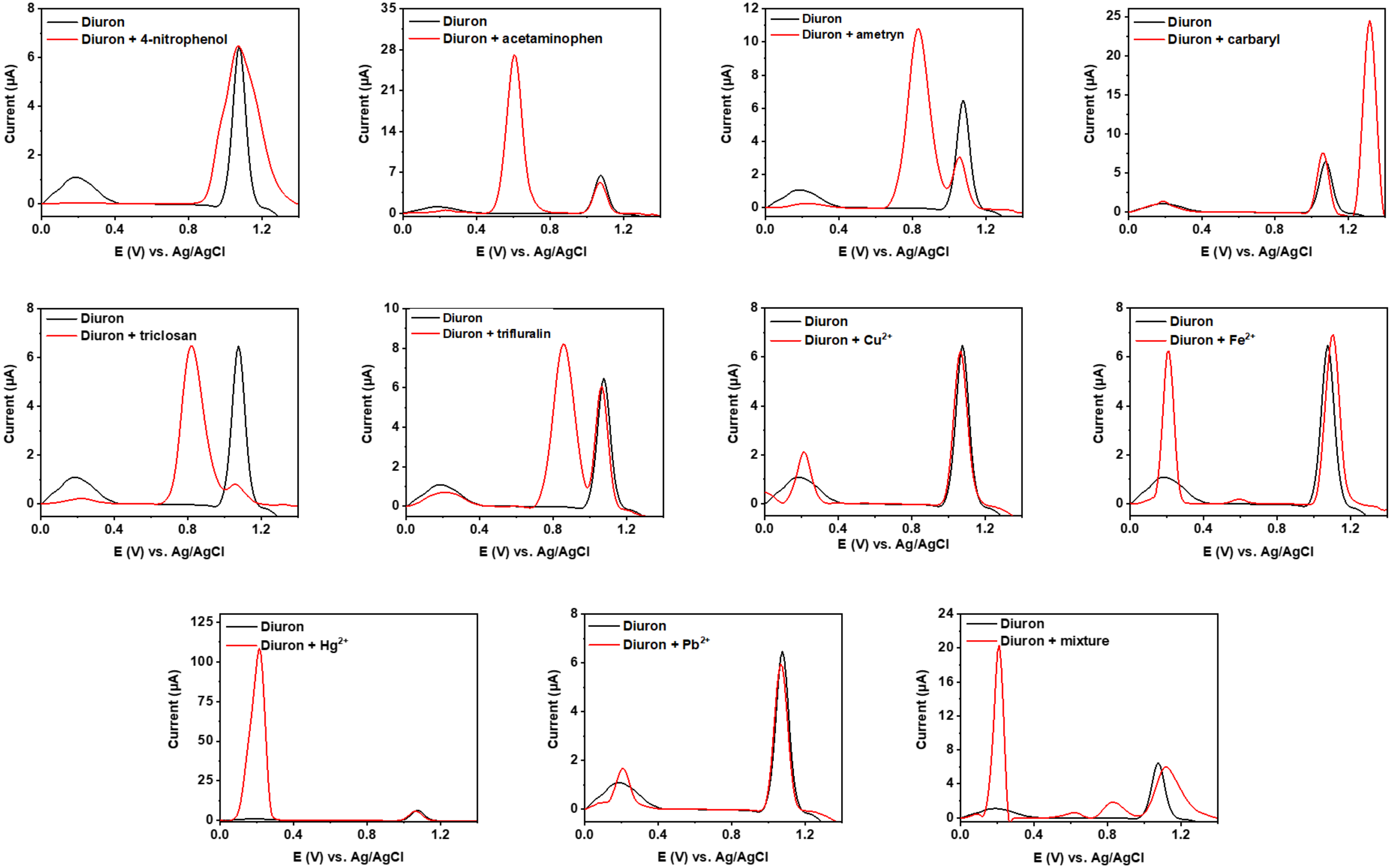

Figure S13. Selectivity study for MCM-41$_{10}$-CPS in BR buffer (pH 3) with diuron (2 mg L^{-1}) in the presence of the following interferents: 4-nitrophenol, acetaminophen, ametryn, carbaryl, triclosan, trifluralin, Cu^{2+}, Fe^{2+}, Hg^{2+}, Pb^{2+}, and their mixture.
